# Human embryonic stem cell-derived exosomes promote pressure ulcer healing in aged mice by rejuvenating senescent endothelial cells

**DOI:** 10.1186/s13287-019-1253-6

**Published:** 2019-05-21

**Authors:** Bi Chen, Yongjin Sun, Juntao Zhang, Qingwei Zhu, Yunlong Yang, Xin Niu, Zhifeng Deng, Qing Li, Yang Wang

**Affiliations:** 1 Department of Orthopedic Surgery, Shanghai Jiao Tong University Affiliated Sixth People’s Hospital, Shanghai, 200233 China; 2 Institute of Microsurgery on Extremities, Shanghai Jiao Tong University Affiliated Sixth People’s Hospital, Shanghai, 200233 China; 3 Department of Neurosurgery, Shanghai Jiao Tong University Affiliated Sixth People’s Hospital, Shanghai, 200233 China

**Keywords:** Senescence, Angiogenesis, Exosomes, Embryonic stem cells, Nrf2

## Abstract

**Background:**

Angiogenesis, as an endogenous repair mechanism, plays crucial roles in wound healing and tissue regeneration. However, this process is impaired in the elderly due to aging-related vascular endothelial dysfunction. This study was aimed to explore the pro-angiogenic effects of exosomes from human embryonic stem cells (ESC-Exos) in aged mice of pressure-induced ulcer model and the underlying mechanism.

**Methods:**

Pressure ulcer wounds were created on the back of d-galactose-induced aging mice. ESC-Exos were locally applied onto the wound beds, with PBS as control. The effects of ESC-Exos on wound healing were analyzed by measuring wound closure rates, histological and immunofluorescence analyses. Then, the anti-aging effect of ESC-Exos on vascular endothelial cells was tested in an in vitro d-galactose-induced HUVEC senescence model.

**Results:**

ESC-Exos could accelerate wound closure and enhance angiogenesis, and the senescence of vascular endothelial cells was significantly ameliorated after ESC-Exos treatment. In vitro, ESC-Exos could rejuvenate the senescence of endothelial cells and recover compromised proliferation, migratory capacity, and tube formation. This recovery was Nrf2-activation-dependent, since cotreatment with Nrf2 inhibitor Brusatol could abolish the rejuvenative effects of ESC-Exos. Further study revealed that miR-200a was highly enriched in ESC-Exos and played a crucial role in ESC-Exos-mediated rejuvenation through downregulating Keap1, which negatively regulates Nrf2 expression.

**Conclusions:**

ESC-Exos ameliorate endothelial senescence by activating Nrf2 and recover aging-related angiogenic dysfunction, thereby accelerating wound healing in aged mice. ESC-Exos might be a natural nano-biomaterial for aging-related diseases therapy.

**Electronic supplementary material:**

The online version of this article (10.1186/s13287-019-1253-6) contains supplementary material, which is available to authorized users.

## Background

Aging, an inevitable biological process, is characterized by the functional decline of tissues and organs [1]. During age process, senescent cells gradually accumulate in multiple tissues and result in impaired homeostasis and regenerative decline, with the expression of senescence-associated β-galactosidase (SA-β-gal), the cyclin-dependent kinase inhibitors P16 and P21, increased oxidative stress levels, and other hallmarks [1, 2]. Effective wound repair requires a coordinated cellular response to restore tissue integrity and function [3]. Angiogenesis, as an endogenous repair mechanism, plays crucial roles in wound healing and tissue regeneration by restoring blood perfusion and delivering nutrients to injured sites [3]. Endothelial cells, a crucial component of the angiogenic process, exhibit impaired function during senescence [4–6], and higher numbers of senescent endothelial cells were demonstrated to occur in aged tissues [4]. Studies on age-dependent differences in wound healing have revealed that wound healing is impaired in old age and insufficient local angiogenesis is considered as a very likely contributor to compromised tissue repair in aged individuals [4, 7]. Thus, therapeutic strategies designed to ameliorate endothelial senescence and impaired angiogenesis may promote wound healing in aged individuals.

As is well known, successful treatment of aging-associated diseases could benefit from stem cell-based therapy, which could regenerate functional integrity and contribute to tissue homeostasis [8]. Adult stem cells of different origins have been reported to alleviate aging-related phenotypes and enhance the functionality of aged individuals [7, 9, 10]. Mesenchymal stem cell transplantation could rejuvenate the aging-related features of senescent cardiomyocytes and osteoarthritic chondrocytes [11, 12]. In addition to adult stem cells, embryonic stem cells (ESCs) promise to be an attractive therapeutic candidate in treating aging-associated diseases due to their unique capacity of proliferating indefinitely, pluripotency, and intrinsic youthful barrier to aging [13]. A study held by Min et al. demonstrated that intramyocardial injection of ESCs could improve myocardial function in aging rats through synergistic angiogenesis and myogenesis [14]. However, the risks of tumorigenesis and immune rejection prevent the large-scale clinical application of stem cell transplantation [13]. Recently, exosomes, as natural nanosized particles secreted from cells, were demonstrated to play crucial roles in intercellular communication. Accumulating studies have revealed that exosomes possess parental stem cell-like pro-regenerative effects against multiple diseases in animal models [15, 16], with no risk of aneuploidy and a lower possibility of immune rejection [17]. We have previously demonstrated that exosomes derived from various adult stem cells aid in cutaneous wound healing [18], bone regeneration [19], and ischemic hindlimb [20]. The mechanisms of tissue repair following exosomes treatment are attributed in part to exosomes-mediated pro-angiogenesis effects at sites of injury [21]. Nevertheless, to date, the therapeutic potential of stem cell-derived exosomes for aging-associated diseases is barely reported. A recent study conducted by Miguel et al. [22] found that exosomes from adipose-derived mesenchymal stem cells ameliorate osteoarthritic osteoblasts’ senescence features induced by interleukin (IL)-1β. We speculate that human ESC-derived exosomes (ESC-Exos) might also have anti-aging function like their parental ESCs. Since qualified ESC-Exos can be obtained infinitely from ESCs, and is convenient for industrial production, it holds great potential in anti-aging therapy.

Pressure ulcer wounds, as one of the most common soft tissue injuries, are particularly hard to heal in aging individuals because of aging-related changes in skin tissue, especially the compromised angiogenic dysfunction. Several attempts have been made to accelerate the wound healing process in aging animal models, but an optimal therapeutic strategy is still lacking [4, 7]. In this study, we verified the therapeutic effects of ESC-Exos in pressure ulcer healing in aged mice. We found that the local application of ESC-Exos significantly accelerated wound healing and promoted angiogenesis at wound sites. Moreover, endothelial cell senescence could be rejuvenated via exosomal miR200a-mediated Kelch-like ECH-associated protein 1 (Keap1) downregulation and the resultant nuclear factor (erythroid-derived 2)-like 2 (Nrf2) activation. These data show for the first time that ESC-Exos facilitate pressure ulcer healing by ameliorating endothelial senescence and impaired angiogenesis.

## Methods

### Cell culture

Human ESCs (hESCs; H9) were cultured on Coring Matrigel hESC-Qualified Matrix (Corning) in mTeSR1 medium (StemCell Technologies). The ALP activity of ESCs was evaluated. Markers of hESCs (Nanog, OCT4, SSEA4, and TRA-1-60) were detected in the hESC colonies using immunostaining.

HUVECs (ScienCell) were cultured in endothelial cell medium (ECM; ScienCell) containing 5% FBS (ScienCell) and 1% endothelial cell growth supplement (ScienCell). In order to induce HUVEC senescence, 10 g/L d-galactose (D-gal; Sigma-Aldrich) was used.

### Isolation and identification of ESC-Exos

The cultured medium from ESCs was collected, and exosomes were isolated by differential centrifugation/ultracentrifugation protocols. Briefly, the obtained medium was centrifuged at 300×*g* for 15 min and 2000×*g* for 30 min to remove dead cells and cellular debris. After centrifugation at 10,000×*g* for 1 h, the supernatant was further ultracentrifuged at 100,000×*g* for 2 h. After the removal of supernatant, the pellet was resuspended in PBS, followed by another ultracentrifugation at 100,000×*g* for 2 h. Finally, pelleted exosomes were resuspended in PBS. Exosomes morphology was observed by TEM (Hitachi H-7650). The size distribution and particle concentration of exosomes were measured using qNano platform (iZON® Science, UK) as described previously [23]. Expression of the exosomal markers CD9 (1:1000; Epitomics), CD63 (1:1000; Epitomics), and TSG-100 (1:1000; Abcam) were analyzed by Western blotting. To detect the purity of the exosomes isolated from ESCs, we measured the expression of cis-Golgi matrix protein GM130 (1:500; Abcam), Actin (1:10,000; Thermo Fisher Scientific), and Lamin A/C (1:1000; Servicebio) in the ESCs and exosomes.

### Aged mouse skin pressure ulcer model and treatment

Animal care and experimental procedures were approved by the Animal Research Committee of the Sixth People’s Hospital at the Shanghai Jiao Tong University. Thirty-six 6- to 8-week-old C57BL/6 male mice were used to evaluate the therapeutic effect of ESC-Exos. The skin aging model was established by daily subcutaneous injection of D-gal (1000 mg/kg) dissolved in 0.9% normal saline (NS) for 8 weeks, and mice in the control group (*n* = 12) were subjected to the same volume of NS. After D-gal or NS administration was finished, mice in the aged model group (*n* = 24) were further classified into two subgroups: the PBS-treated (Aged-PBS) group (*n* = 12) and the ESC-Exos-treated (Aged-Exos) group (*n* = 12).

Then, pressure ulcers were created on the back of each mouse as previously reported [24, 25]. Briefly, mice were anesthetized by intraperitoneal injection of 50 mg/kg pentobarbital sodium (Sigma-Aldrich), and the dorsal skin was gently pulled up and pinched between two circular 12-mm diameter magnets for 12 h, followed by a rest period of 12 h, as one ischemia reperfusion (IR) cycle. Seven days after three IR cycles, two pressure ulcer wounds were established [25]. ESC-Exos (1 × 10^10^ particles/100 μL) were locally applied using pipettes onto the wound beds of mice one time per day in the Aged-Exos group, while ulcer wounds in the Aged-PBS and control groups were treated with sterile PBS. The ulcer wound area was observed daily during 3-week experimental periods and imaged at indicated time points. The wound closure rate was calculated by *ImageJ* software (National Institute of Health, USA) as follows: wound closure (%) = (*A*_0_ − *A*_t_)/*A*_0_ × 100, where *A*_0_ is the initial wound area and *A*_t_ is the wound area at the indicated times.

### ESC-Exos permeation on wound beds

Exosomes were labeled with a green fluorescent dye (DIO; Life Technologies) according to the manufacturer’s recommended instructions. Labeled exosomes were locally applied onto the wound beds, and wound sites without labeled exosomes administration were set as control (*n* = 3). The skin samples were fixed with 4% paraformaldehyde at indicated time point 24 h (*n* = 3), followed by OCT-compound (Tissue-Tek, Sakura, Japan) embedment. After the nuclei were stained with Dapi, the sections were viewed using fluorescence microscopy and imaged.

### Histological analysis

The skin specimens were fixed in 4% paraformaldehyde, dehydrated through a series of graded ethanol, embedded in paraffin, and then cut into 5-μm-thick sections. For histological observation, the sections were stained with hematoxylin and eosin (H&E) and the scar width was measured. Masson’s trichrome staining was used to determine the degree of collagen maturity.

### Immunohistochemistry and immunofluorescence staining

For immunohistochemistry analysis, sections were rehydrated, blocked, and incubated with primary anti-CD31 (1:100; Abcam) antibodies overnight at 4 °C, followed by the biotinylated secondary antibody and avidin-biotin-peroxidase complex at room temperature. The DAB substrate was used to visualize the stained sections. Finally, the sections were counter-stained with hematoxylin.

For immunohistochemistry staining, sections were incubated with primary antibodies against the following proteins: P16 (1:100; Abcam), CD31 (1:100; Abcam), α-SMA (1:100; Cell Signaling Technology), and Nrf2 (1:100; Abcam). After treating the sections with Alexa Fluor 488- and Alexa Fluor 594-conjugated secondary antibodies, DAPI (Beyotime Biotechnology) was used to stain the nuclei.

For immunocytochemistry, fixed cells were stained with primary antibodies against Ki67 (1:200; Cell Signaling Technology) and P16 (1:200; Abcam), followed by secondary antibody conjugated to Alexa Fluor 594 (Life Technologies). Nuclei were stained with DAPI. Quantification of the number of positively stained cells was performed using the *ImageJ* software.

### Vascularization assessment

Mice were killed and perfused with Microfil (Microfil MV-122; Flow Tech, Carver, MA, USA). Then, the samples were analyzed by micro-CT (Skycan 1176; Burker), and three-dimensional images were reconstructed with the CTVol program (Bruker). The number of blood vessels was determined with the *ImageJ* software.

### Measurement of oxidative stress level

Skin tissue and cell samples were lysed, and the supernatant was collected. The levels of SOD, MDA, CAT, and GSH-PX were determined by relevant commercial kits (Nanjing Jiancheng Bioengineering Institute, China).

For intracellular ROS measurement, HUVECs were incubated with 10 μmol/L DCFH-DA (Beyotime Biotechnology) in an incubator at 37 °C for 20 min, and the accumulation of ROS in cells was viewed using fluorescence microscopy and imaged.

### In vitro effects of ESC-Exos on HUVEC senescence

Senescent HUVECs were incubated with 1 × 10^10^ particles/mL for different time periods (Aged-Exos Group) while HUVECs in the control groups were treated with an equal volume of exosome diluent (PBS) or “young” HUVECs (without D-gal treatment). For testing the role of Nrf2 activation in ESC-Exos-mediated rejuvenation of senescent HUVECs, the cells were cultured under different treatment conditions: (1) Aged group (treated with vehicle), (2) Aged-Exos group (treated with 1 × 10^10^ particles/mL ESC-Exos), and (3) Aged-Exos-Brusatol (Sigma-Aldrich) group (co-treated with 1 × 10^10^ particles/mL ESC-Exos and 40 nM Brusatol).

### Exosomes uptake assay

Exosomes were labeled with a green fluorescent dye (DIO; Life Technologies). Then, aged HUVECs were incubated with DIO-labeled ESC-Exos for 12 h, and nuclei were stained with DAPI.

### miRNA inhibitor transfection

ESCs at 70% confluence were transfected with 100 nM miR-200a antagomir (RiboBio, China) and antagomir negative control using Lipofectamine RNAiMAX according to the manufacturer’s procedures. The transfected cells were cultured with for 48 h. The exosomes were then isolated from the culture supernatant. Then, senescent HUVECs were cultured in 6-well plates with different treatment conditions: (1) Aged group (aged HUVECs treated with vehicle), (2) Aged-NCI-Exos group (NCI-ESC-Exos, exosomes isolated from antagomir negative control treated ESCs), and (3) Aged-200aI-Exos group (200aI-ESC-Exos, exosomes isolated from miR-200a antagomir treated ESCs). After the treatment, the downstream experiments were performed.

### Dual-luciferase reporter assay

The fragment of wild-type (WT) Keap1 3′-UTR (Keap1-WT) containing predicted miR-200a target sites was amplified by PCR. The fragments including the 3′-UTR WT regions of Keap1 were cloned into the pMir-Glo (Promega, USA) vector. The mutant Keap1 3′-UTR (Keap1-MUT) was generated by mutating the binding sites for miR-200a using Gene Mutation Kit (Takara, Japan). Then, the Keap1-WT or Keap1-MUT plasmid was co-transfected with miR-200a mimics into HUVECs. The miR-NC mimics were set as the negative control. After 48 h post-transfection, cells were harvested. Firefly and Renilla luciferase activities were measured using the Dual-luciferase Reporter Assay System kit (Promega, USA) following the manufacturer’s protocol.

### Senescence-associated β-galactosidase staining

SA-β-gal staining of HUVECs was performed using an SA-β-gal staining kit (Beyotime Biotechnology). Briefly, the cells were fixed and then stained with SA-β-gal staining solution for 24 h at 37 °C (without CO_2_). The cells were observed using a phase-contrast microscope and then imaged. The ratio of positive cells was determined by counting the blue cells and dividing by the total number of observed cells.

### Western blotting

Cells were harvested using RIPA lysis buffer supplemented with protease inhibitor cocktail (Roche) to obtain the whole protein lysate. In the case of nuclear protein extraction, the Nuclear and Cytoplasmic Protein Extraction Kit (Beyotime Biotechnology) was used following the manufacturer’s protocols. Protein extracts were separated by sodium dodecyl sulfate-polyacrylamide gel electrophoresis (SDS-PAGE) and transferred to polyvinylidene fluoride (PVDF) membranes. The membranes were blocked with 5% non-fat milk for 1 h and incubated overnight at 4 °C with antibodies against the following proteins: P16 (1:1000; Abcam), P21 (1:1000; Abcam), Nrf2 (1:1000; Abcam), Keap1 (1:1000; Cell Signaling Technology), HO1 (1:1000; Cell Signaling Technology), Actin (1:10,000; Thermo Fisher Scientific), and Histone H3 (1:1000, Cell Signaling Technology). Membranes were then incubated with HRP-conjugated anti-rabbit (1:1000) or anti-mouse (1:4000) secondary antibodies (Cell Signaling Technology).

### Proliferation assay

The proliferation of HUVECs was measured using a cell counting kit-8 kit (CCK8; Dojindo Melocular Technologies). HUVECs from different treatment groups (3 × 10^3^ cells per well) were seeded into 96-well plates. On days 1, 2, 3, 4, and 5, 10 μL CCK8 reagent was added to the culture medium (100 μL per well), and the absorbance of each well was observed at 450 nm by a microplate reader (Bio-Rad 680, Hercules, USA).

### Wound healing assay

A culture-insert (ibidi GmbH, Germany) was used to evaluate the migratory behavior of HUVECs. Briefly, cells were seeded and incubated at 37 °C. Then, the culture-insert was removed leaving a cell-free gap (“defined wound”). The migration of HUVECs into the “wound area” was imaged and measured after 24 h. The closure area of the wound was calculated as follows: Migration area (%) = (*A*_0_ − *A*_n_)/*A*_0_ × 100, where *A*_0_ represents the initial wound area and *A*_n_ represents the remaining wound area at the measured time point.

### Transwell migration assay

A total of 4 × 10^4^ cells/well were suspended in low serum (0.5% FBS) ECM medium and seeded into the upper chamber of 24-well transwell plates (Corning, USA) with 12-μm pore size. Then, the lower chamber was added with complete ECM medium (containing 5% FBS). After 18 h, cells were stained with 0.5% crystal violet; the migrated cells were imaged using an optical microscope.

### Tube formation

A total of 1.2 × 10^5^ HUVECs/well were added to polymerized Matrigel (BD Biosciences) in 24-well plates. After incubation at 37 °C for 12 h, the tube formation was observed, and the total branching points and total tube length were measured by the *ImageJ* software.

### Quantitative real-time PCR analysis

Exosomal miRNAs were isolated by using the Exosome RNA Purification Kit (Qiagen), and the reverse transcription reactions of miRNAs were performed using the 4× Reverse Transcription Master Mix kit (EZBioscience). The stem-loop RT primers are listed in Additional file 7: Table S1. The qRT-PCR analyses of miRNAs were carried out with FastStart Universal SYBR Green Master (Roche); the gene-specific primers are listed in Additional file 7 Table S1.

### Statistical analysis

All of the results are expressed as the means ± SEM. Statistical analysis was performed using a Student’s *t* test (for single comparisons) or analysis of variance (ANOVA) for multiple comparisons. A value of *P* < 0.05 was used to indicate statistical significance.

## Results

### Characterization of ESCs and ESC-derived exosomes

**Fig. 1:**
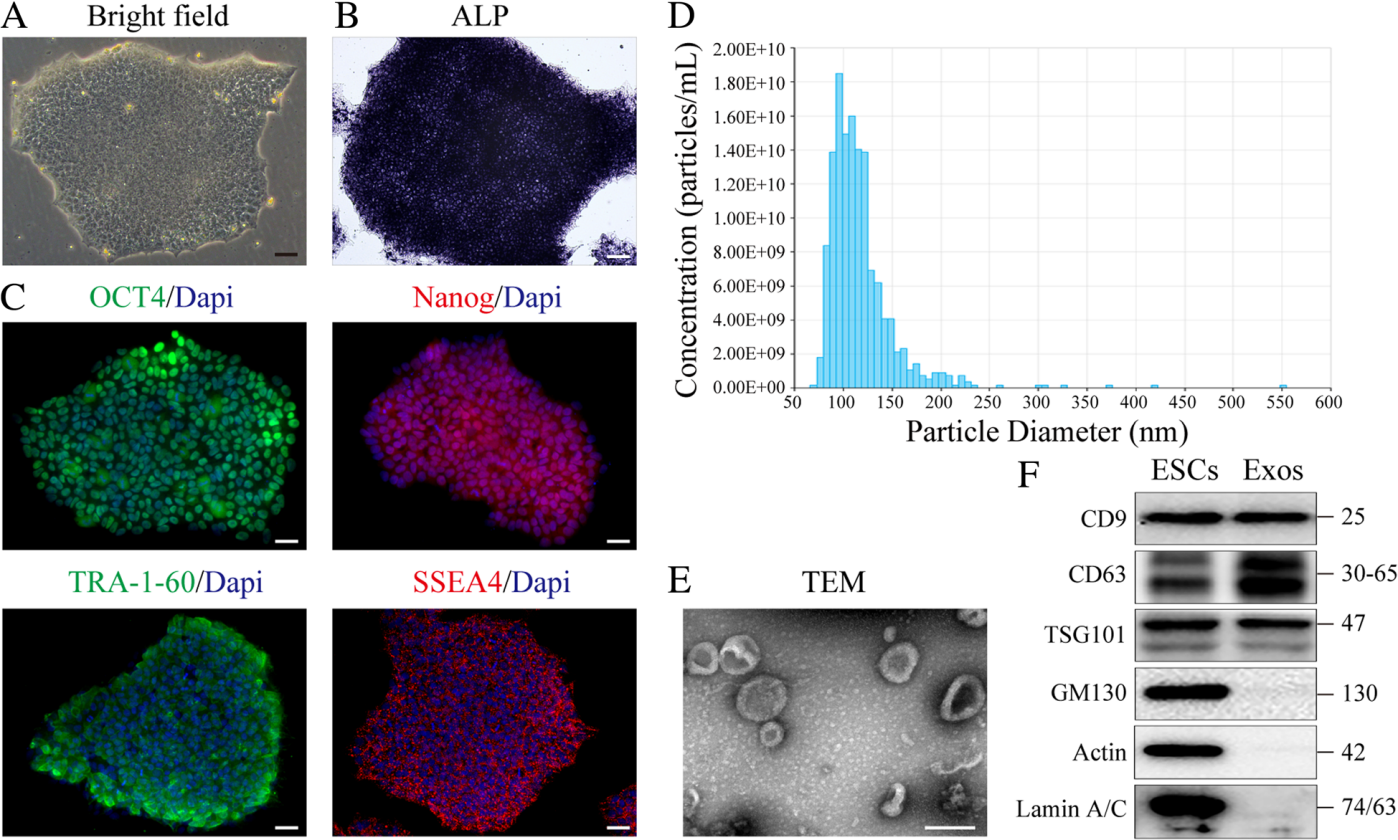
Characterization of ESCs and ESC-derived exosomes. **a** ESC colonies morphology. Scale bar, 100 μm. **b** ALP staining of ESCs. Scale bar, 100 μm. **c** IF staining analysis of pluripotency-related markers of ESCs. Scale bar, 50 μm. **d** Particle size distribution of ESC-Exos measured by qNano analysis. **e** Morphology of ESC-Exos observed by TEM. Scale bar, 100 nm. **f** Western blotting showed the presence of exosomal markers including CD9, CD63, and TSG101, but exosomes were negative for GM130, Actin, and Lamin A/C

Immunostaining analysis was used to measure the expression levels of cell-specific antigens in ESC colonies. As shown in Fig. 1a–c, ESC colonies expressed pluripotency-related markers, including OCT4, Nanog, TRA-1-60, and SSEA4, as well as alkaline phosphatase (ALP). Exosomes were isolated from the cultured medium of ESCs. Next, qNano analysis was performed to identify the ESC-Exos, showing that the size distribution of most exosomes was in the range of 50–150 nm (Fig. 1d). By transmission electron microscopy (TEM) imaging (Fig. 1e), the vesicles with characteristic cup-shaped morphology were observed. By Western blot analysis, we demonstrated the presence of exosomal markers, including CD9, CD63, and TSG101, but not of the cis-Golgi matrix protein GM130, Actin, and Lamin A/C (Fig. 1f), which means no contamination of cellular components in isolated exosomes.

### d-Galactose-induced skin aging model

The animal aging model was generated by chronic administration of d-galactose (D-gal). As shown in Additional file 1: Figure S1A, D-gal-treated mice exhibited obvious skin aging phenotypes compared with mice in the control group. H&E staining showed the reduced dermis and muscle thickness in D-gal-treated skin while no significant difference was observed in the thickness of other layers of the skin (Additional file 1: Figure S1B–C). Moreover, immunofluorescence (IF) staining against P16 showed that the number of P16-positive cells significantly increased in the group of D-gal-treated mice compared to that in the control group (Additional file 1: Figure S1D), which is consistent with a previous report [26]. Considering that increased oxidative stress is a key mechanism underlying D-gal-induced aging, MDA, SOD, CAT, and GSH-Px levels were measured in skin tissues from each group. We found that MDA level was significantly increased in D-gal-treated mice, while the activity of SOD, CAT, and GSH-Px levels were obviously decreased (Additional file 1: Figure S1E). All of these changes are consistent with the performance during natural aging [7, 26]. These results suggest that the D-gal-induced skin aging model has been successfully established.

### ESC-Exos promote pressure ulcer wound healing in aged mice

**Fig. 2:**
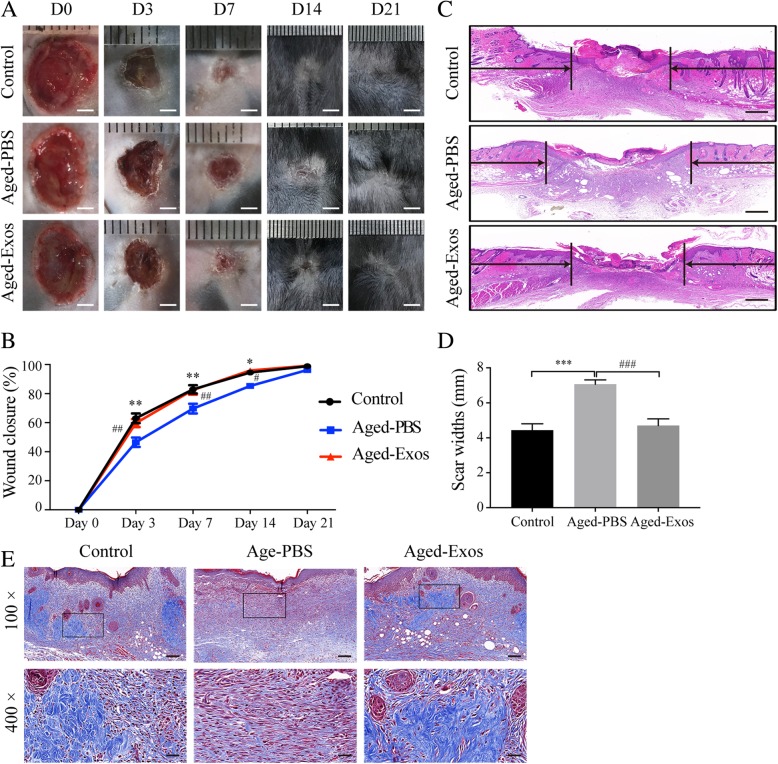
ESC-Exos promoted pressure ulcer wound healing in aged mice. **a** Gross view of wounds treated with ESC-Exos or PBS in aging mice and wounds with PBS in the young control group, at days 3, 7, 14, and 21 post-wounding. Scale bar, 2 mm. **b** The rate of wound closure of three groups. *n* = 6 per group. **P < 0.01; *P < 0.05 Aged-PBS versus control group; ^##^*P* < 0.01; ^#^*P* < 0.05 Aged-Exos versus Aged-PBS group. **c** H&E staining of wound sections from three groups at 7 days after initial treatment. The black arrows indicate the edges of the scar. *n* = 3 per group. Scale bar, 500 μm. **d** Quantification of the scar widths. *n* = 3 per group. ***P < 0.001 Aged-PBS versus control group; ^###^*P* < 0.001 Aged-Exos versus Aged-PBS group. **e** Masson’s trichrome staining of wound sections from three groups. *n* = 3 per group. Scale bar, 100 μm (top) or 25 μm (bottom)

We first determined whether ESC-Exos could permeate through pressure ulcer wound beds, and representative permeation imaging of wound beds were collected at 24 h after DIO-labeled ESC-Exos local administration. The results revealed that ESC-Exos could be reached deep dermis of wound beds after local application (Additional file 1: Figure S1F). Then, to evaluate the effects of ESC-Exos on cutaneous wound healing in aged mice, pressure ulcer wounds were created on the back of aged mice, followed by daily local application of ESC-Exos (Aged-Exos group) or an equal volume of PBS (Aged-PBS group), with wounds in young mice without exosomes treatment as control. As shown in Fig. 2a, b, the wounds in the Aged-PBS group showed significantly delayed healing compared to those in the control group, as determined wound areas’ measurement at days 3, 7, and 14 after initial treatment. The local application of exosomes could accelerate wound healing in aged mice which is comparable to that of the young control group. By day 21, all the wounds achieved similar closure. H&E staining on day 7 revealed the compromised regeneration of the neo-epidermis and dermis in the Aged-PBS group compared with that in the control group, and enhanced regeneration was observed in the Aged-Exos group (Fig. 2c–d). The collagen deposition in Aged-Exos group as shown by Masson’s trichrome staining increased compared with that of the Aged-PBS group (Fig. 2e). These data indicate that local application of ESC-Exos results in accelerated pressure ulcer healing in aged mice, which is comparable to that in young mice.

### ESC-Exos enhance angiogenesis and ameliorate vascular endothelial cell senescence in the wound sites of mice

**Fig. 3:**
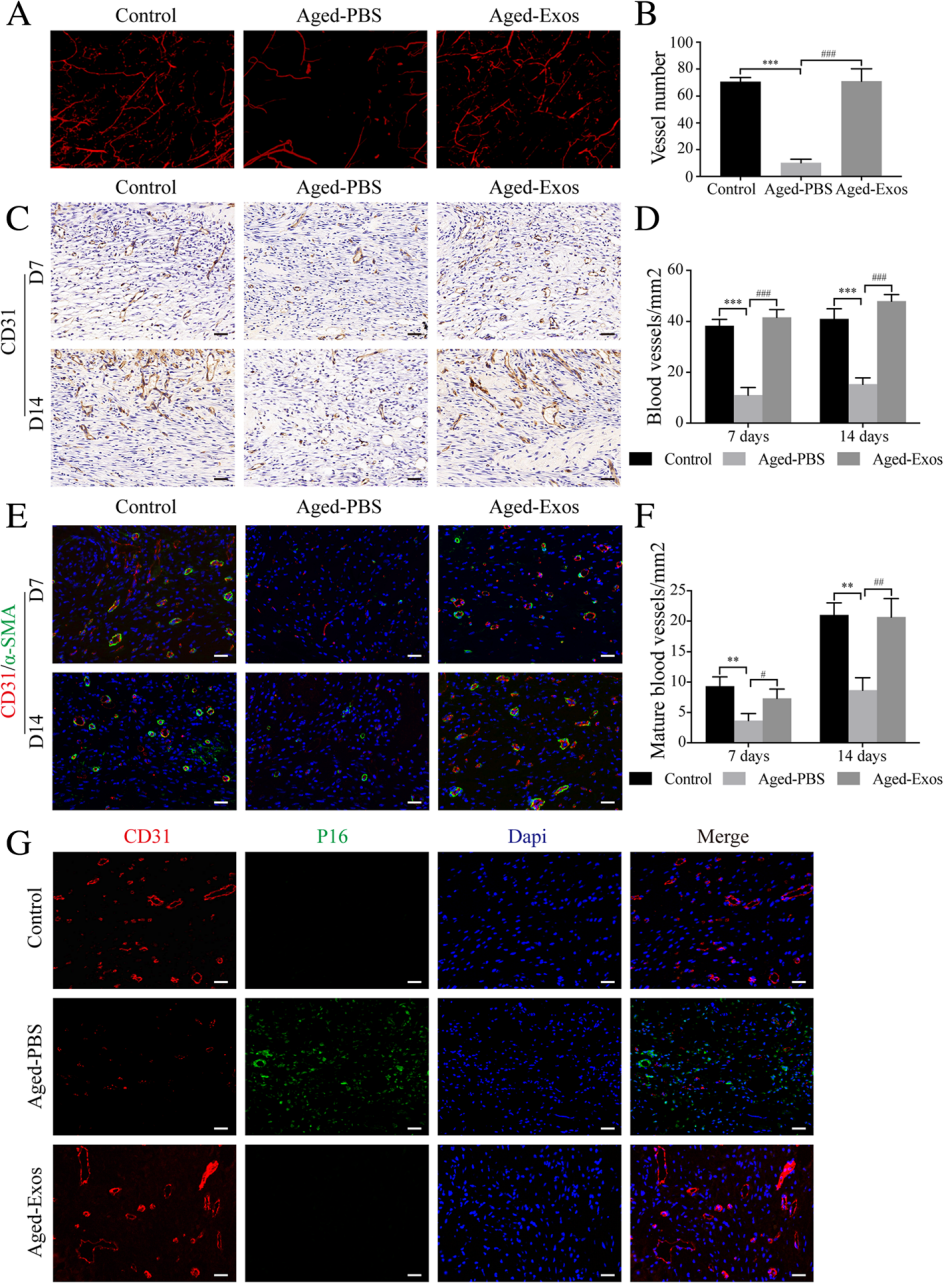
ESC-Exos enhanced angiogenesis and ameliorated vascular endothelial cell senescence in the wound sites of aged mice. **a** Micro-CT images of blood vessel formation in pressure ulcer wounds from the indicated groups at 14 days after initial treatment. *n* = 3 per group. **b** Quantification analysis of the number of blood vessels. *n* = 3 per group. ***P < 0.001 Aged-PBS versus control group; ^###^*P* < 0.001 Aged-Exos versus Aged-PBS group. **c** IHC staining of CD31 of wound sections at 7 and 14 days after initial treatment. *n* = 3 per group. Scale bar, 50 μm. **d** Statistical results from **c**. *n* = 3 per group. ***P < 0.001 Aged-PBS versus control group; ^###^*P* < 0.001 Aged-Exos versus Aged-PBS group. **e** IF staining against CD31 and α-SMA. Endothelial cells (CD31), smooth muscle cells (α-SMA), and cell nuclei were stained red, green, and blue, respectively, at 7 and 14 days after initial treatment. *n* = 3 per group. Scale bar, 50 μm. **f** Statistical results from **e**. *n* = 3 per group. **P < 0.01 Aged-PBS versus control group; ^##^*P* < 0.01; ^#^*P* < 0.05 Aged-Exos versus Aged-PBS group. **g** IF staining against CD31 and P16. Endothelial cells (CD31), senescent cells (P16), and cell nuclei were stained red, green, and blue, respectively, at 14 days after initial treatment. *n* = 3 per group. Scale bar, 50 μm

Vasculature formation in wound sites has been recognized as a crucial element for cutaneous wound healing [3]. We then evaluated the role of ESC-Exos in angiogenesis at the wound site of aged mice. Fourteen days after initial treatment, the formation of blood vessels was evaluated by micro-CT. The reconstructed three-dimensional images showed that at wound defects in the Aged-PBS group, the vessel density is much lower than that in the control group, while exosome treatment recovered the vessel density to the extent comparable to that in the young control group (Fig. 3a, b). Immunohistochemistry (IHC) staining for CD31 and IF staining against CD31 and α-SMA confirmed these results. As shown in Fig. 3c, d, similar to the young control group, the Aged-Exos group exhibited significantly higher vessel densities both at day 7 and 14 compared to that in the Aged-PBS group. Moreover, ESC-Exos treatment increased the number of mature vessels, as determined by the CD31 and α-SMA double-positive cells (Fig. 3e, f). These results suggest that ESC-Exos could enhance angiogenesis and promote vessel maturation in aged mice.

Previous studies have demonstrated that senescence of endothelial cells lead to impaired angiogenesis, while rejuvenation of these senescent cells can ameliorate the aging-related angiogenic dysfunction, promote neo-vascularization, and enhance wound healing in aged skin [4, 6]. Therefore, we observed whether ESC-Exos treatment could rejuvenate senescent vascular endothelial cells at the wound site in aged mice by co-staining against CD31 and p16. A large number of senescent vascular endothelial cells were identified at wound beds in the Aged-PBS group, while endothelial senescence was barely observed in the young control group. ESC-Exos treatment significantly reduced the number of senescent endothelial cells (Fig. 3g). These results indicate that ESC-Exos can ameliorate endothelial senescence and recover aging-related angiogenic dysfunction, thereby accelerating pressure ulcer healing in aged mice.

### ESC-Exos can ameliorate endothelial senescence and aging-related angiogenic dysfunction in vitro

**Fig. 4:**
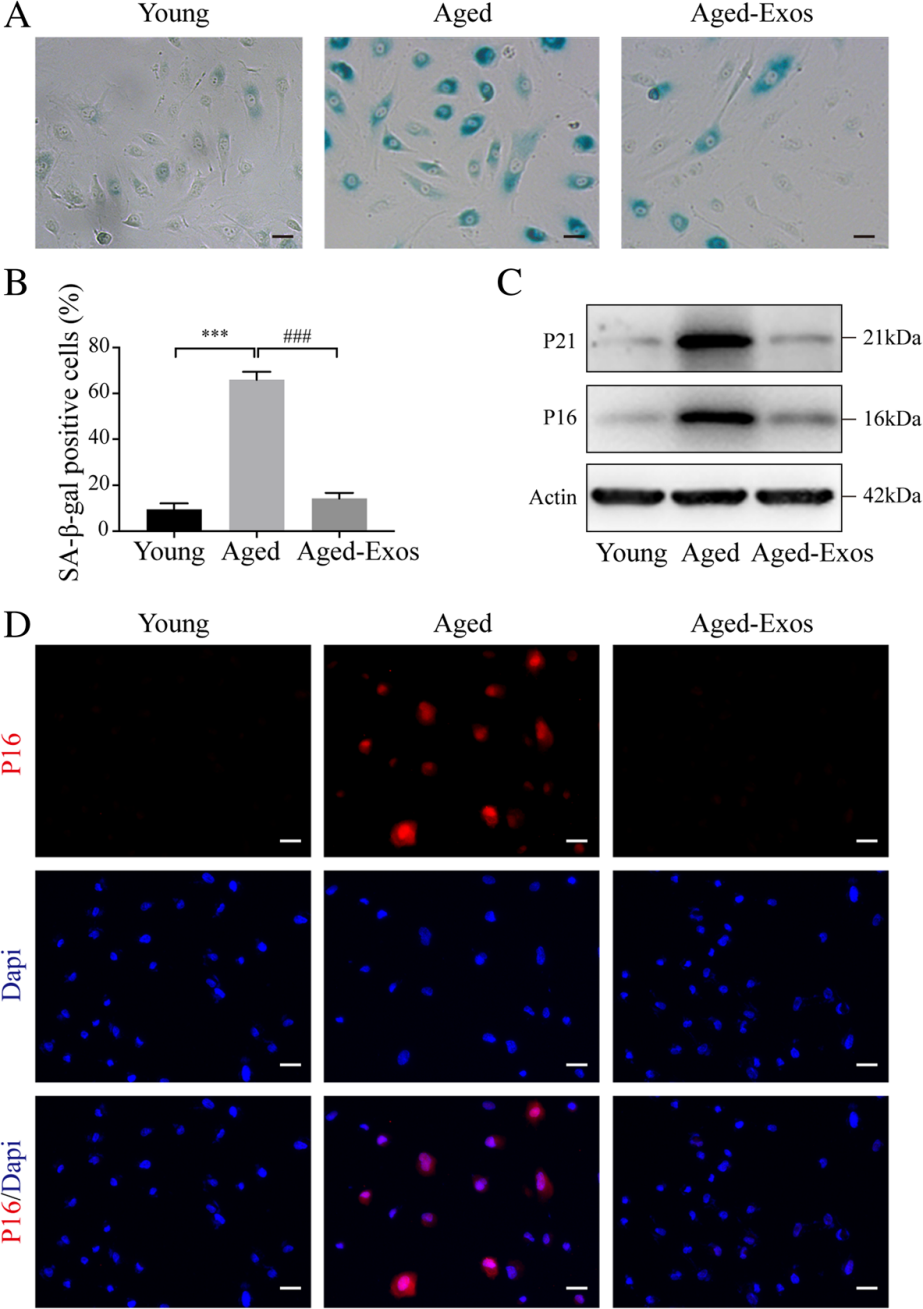
ESC-Exos can ameliorate endothelial senescence induced by D-gal in vitro. HUVECs were treated with 10 g/L D-gal to induce senescence, and aged HUVECs were then treated with 1 × 10^10^ particles/mL ESC-Exos or PBS, while young HUVECs (without D-gal treatment) were set as the control. **a** SA-β-gal staining. SA-β-gal-positive cells are shown in blue when observed under an optical microscope. *n* = 3 per group. Scale bar, 50 μm. **b** Statistical results from (**a**). *n* = 3 per group. ***P < 0.001 Aged versus Young group; ^###^*P* < 0.001 Aged-Exos versus Aged group. **c** Western blotting analysis of P16 and P21 expression. *n* = 3 per group. **d** IF staining was performed to assess the expression level of P16 (red). P16-positive cells were significantly reduced in number after ESC-Exos treatment. *n* = 3 per group. Scale bar, 50 μm

Next, we established an in vitro model of senescence through D-gal treatment of human umbilical vein endothelial cells (HUVECs). The results show that the number of SA-β-gal-positive cells (Fig. 4a, b), as well as the P16 and P21 protein expression levels (Fig. 4c), significantly increased after D-gal treatment (Aged group) compared to that in the young control group. IF staining against P16 demonstrated that the number of P16-positive cells increased (Fig. 4d). To examine the effect of ESC-Exos on endothelial senescence, we first determined whether ESC-Exos could be internalized into aged HUVECs. The result shows that the green fluorescent dye (DIO)-labeled ESC-Exos were transferred to HUVECs after incubation for 12 h (Additional file 2: Figure S2A). Then, the senescent HUVECs were incubated with 1 × 10^10^ particles/mL ESC-Exos. The number of SA-β-gal-positive cells gradually decreased in an incubation time-dependent manner (Additional file 2: Figure S2B). Then, 9 days’ incubation of ESC-Exos was used in the follow-up experiment. As shown in Fig. 4a, b, ESC-Exos treatment significantly reduced the number of SA-β-gal-positive cells. Western blot analysis showed that the P16 and P21 protein expression levels were obviously downregulated after ESC-Exos treatment (Fig. 4c). The number of P16-positive cells also decreased as shown by IF staining (Fig. 4d). These results indicate that endothelial cell aging phenotypes induced by D-gal can be reversed by ESC-Exos treatment.

**Fig. 5:**
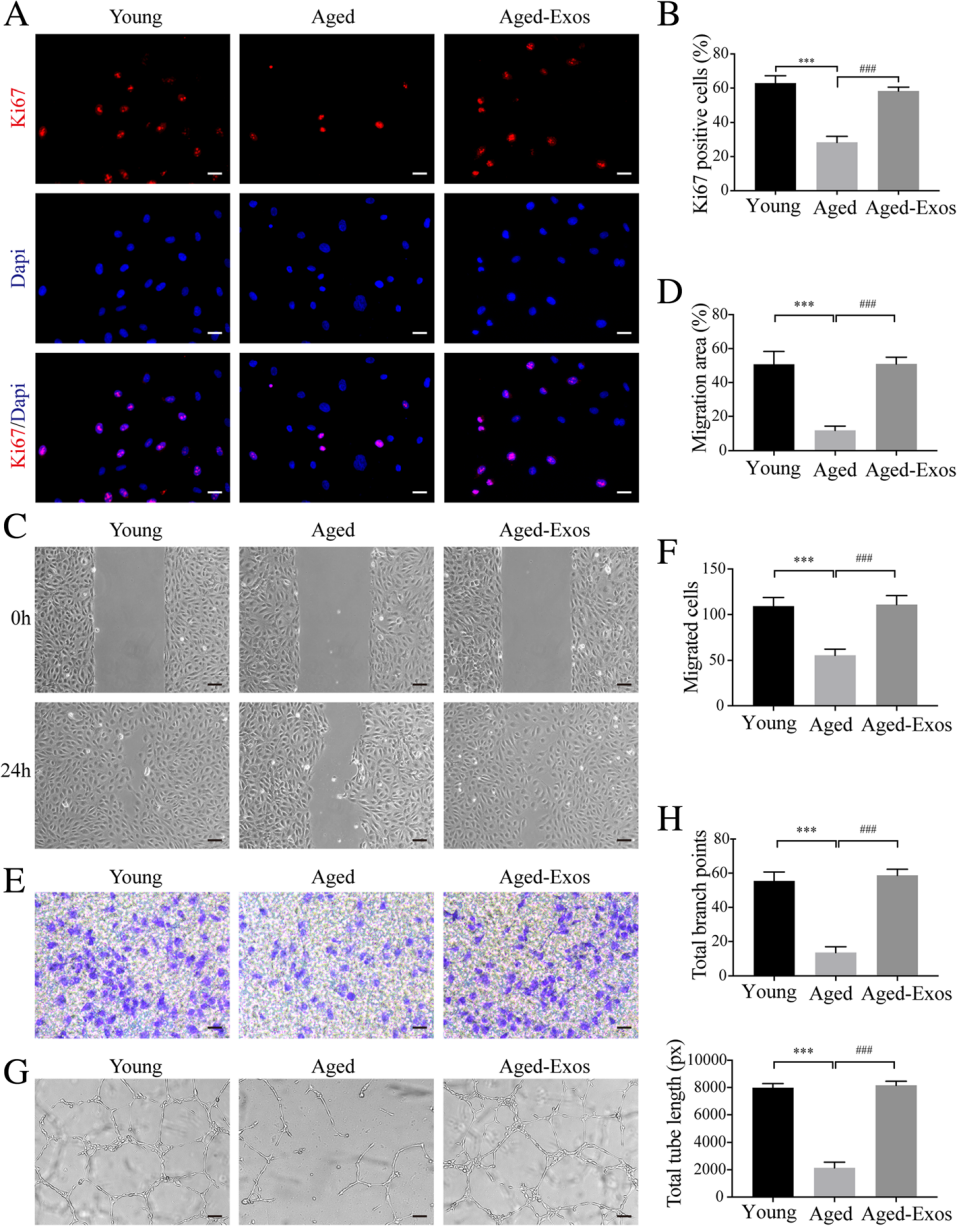
ESC-Exos can ameliorate aging-related angiogenic dysfunction induced by D-gal. HUVECs were treated with D-gal to induce senescence, followed by treatment with ESC-Exos or PBS, and young HUVECs were set as the control. **a** IF staining against Ki67 was performed to assess the proliferative capacity of HUVECs. *n* = 3 per group. Scale bar, 50 μm. **b** Quantification of the number of ki67-positive cells in (**a**). *n* = 3 per group. ***P < 0.001 Aged versus Young group; ^###^*P* < 0.001 Aged-Exos versus Aged group. Wound healing assay (**c**, **d**) (scale bar, 100 μm) and transwell assay (**e**, **f**) (scale bar, 100 μm) revealed that ESC-Exos treatment could recover the compromised migratory ability of aged HUVECs. *n* = 3 per group. ***P < 0.001 Aged versus Young group; ^###^*P* < 0.001 Aged-Exos versus Aged group. **g** Representative images of the tube formation assay in young HUVECs or aged HUVECs after treatment with ESC-Exos or PBS. *n* = 3 per group. Scale bar, 200 μm. **h** Quantitative analyses of the total tube length and branch points. *n* = 3 per group. ***P < 0.001 Aged versus Young group; ^###^*P* < 0.001 Aged-Exos versus Aged group

Given that the angiogenic activities of endothelial cells are compromised in the aging process [5], we next detected the effect of ESC-Exos treatment on the angiogenic phenotype of senescent HUVECs. IF staining against Ki-67 and CCK8 assay were performed to evaluate the proliferative ability of HUVECs. The results showed that D-gal treatment significantly impaired the proliferative potential compared to the untreated HUVECs (the young group), while ESC-Exos incubation recovered the proliferation of D-gal-induced aged endothelial cells (Fig. 5a, b, Additional file 3: Figure S3). The wound healing assay (Fig. 5c, d) and transwell assay (Fig. 5e, f) revealed that the migratory capacity of senescent HUVECs was compromised and could be restored after ESC-Exos treatment. The tubule formation of senescent HUVECs was also recovered after ESC-Exos treatment (Fig. 5g, h). These results suggest that ESC-Exos could rejuvenate senescent endothelial cells and restore angiogenic capacity.

### ESC-Exos rejuvenate senescent endothelial cells through activating Nrf2

**Fig. 6:**
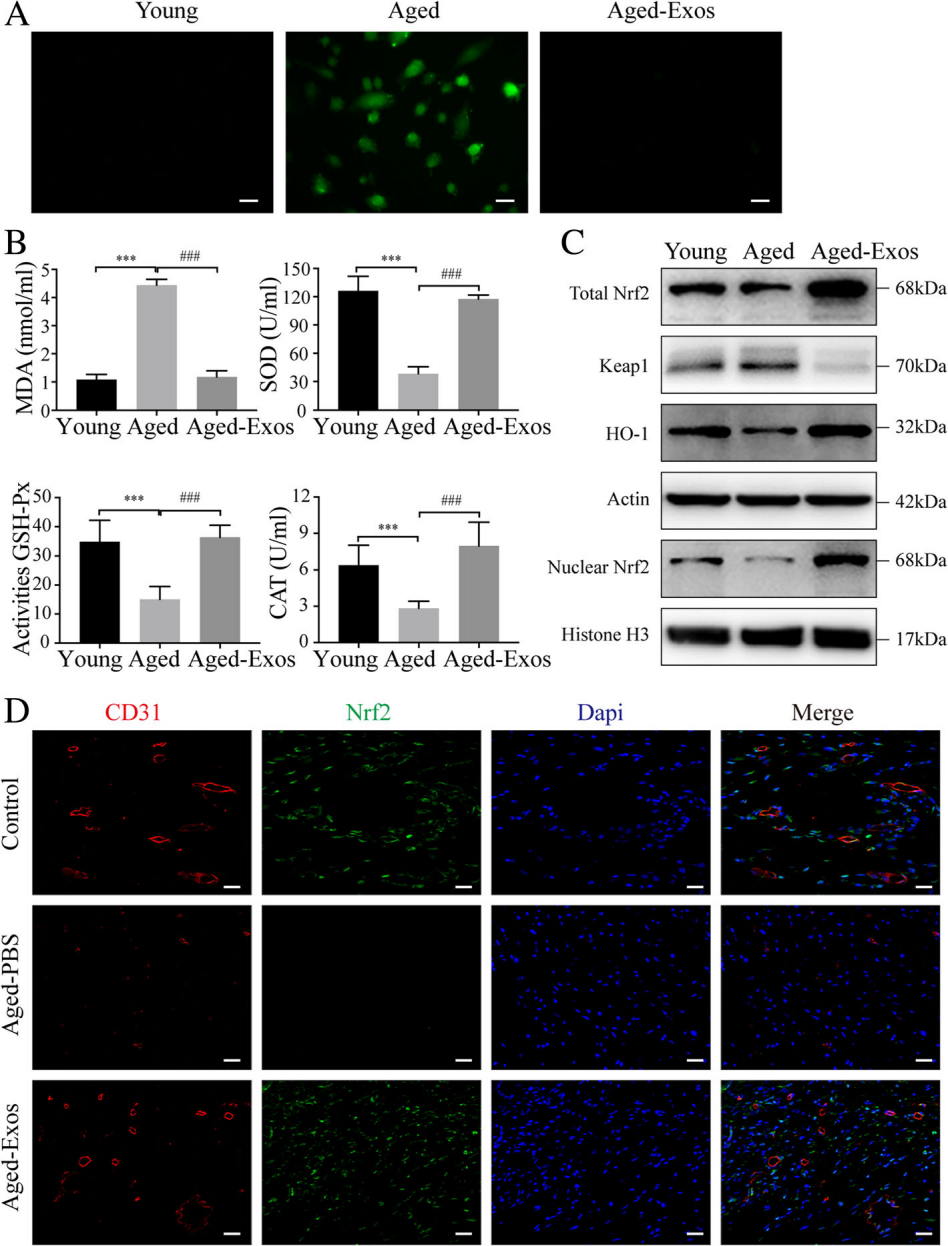
ESC-Exos could reduce oxidative stress and enhance the activity of the endogenous Nrf2 anti-oxidative system. **a** Intracellular ROS levels were determined by green fluorescent intensity after cells were labeled with DCFH-DA. *n* = 3 per group. Scale bar, 50 μm. **b** Oxidative stress levels were evaluated by the activity measurement of MDA, SOD, GSH-Px and CAT. *n* = 3 per group. ***P < 0.001 Aged versus Young group; ^###^*P* < 0.001 Aged-Exos versus Aged group. **c** Protein expression levels of Nrf2 and HO1 in indicated groups. *n* = 3 per group. **d** IF staining against CD31 and Nrf2. Endothelial cells (CD31), Nrf2-positive cells, and cell nuclei were stained red, green, and blue, respectively, at 14 days after initial treatment. The white arrows indicate the Nrf2^+^ vascular endothelial cells. *n* = 3 per group. Scale bar, 50 μm

Increased oxidative stress, as a major characteristic of aging, has been implicated in vascular endothelial senescence [27]. As shown in Fig. 6a, D-gal treatment resulted in an increase of reactive oxygen species (ROS) level in HUVECs, while this effect was almost abolished after ESC-Exos incubation. And the levels of MDA, SOD, CAT, and GSH-Px were also measured. The results showed that D-gal treatment greatly increased the levels of MDA and reduced the activities of these anti-oxidation-related molecules SOD, CAT, and GSH, while ESC-Exos treatment significantly reversed these changes (Fig. 6b). These results suggest that ESC-Exos could reduce oxidative stress by enhancing the activity of the endogenous anti-oxidative system.

Nrf2, as a transcription factor, regulates the expression of multiple ROS detoxifying and antioxidant genes [28]. Recent studies reveal that Nrf2 activity declines in several models of aging or chronic oxidative stress, and upregulation of Nrf2 signaling can ameliorate aging-associated dysfunction [29–32]. Therefore, we tested whether Nrf2 was involved in the effects of ESC-Exos on senescent endothelial cells. Our results showed that total and nuclear Nrf2 protein declined obviously during senescence, and the expression of HO1 (a downstream target of Nrf2) was also decreased in aged HUVECs (Fig. 6c). ESC-Exos treatment could restore the expression of Nrf2 and HO1 (Fig. 6c). And IF staining against CD31 and Nrf2 was performed in vivo 14 days after initial treatment. As shown in Fig. 6d, a large number of Nrf2-positive endothelial cells were identified at wound beds in control and Aged-Exos groups, while Nrf2-positive cells were barely observed in the Aged-PBS group.

**Fig. 7:**
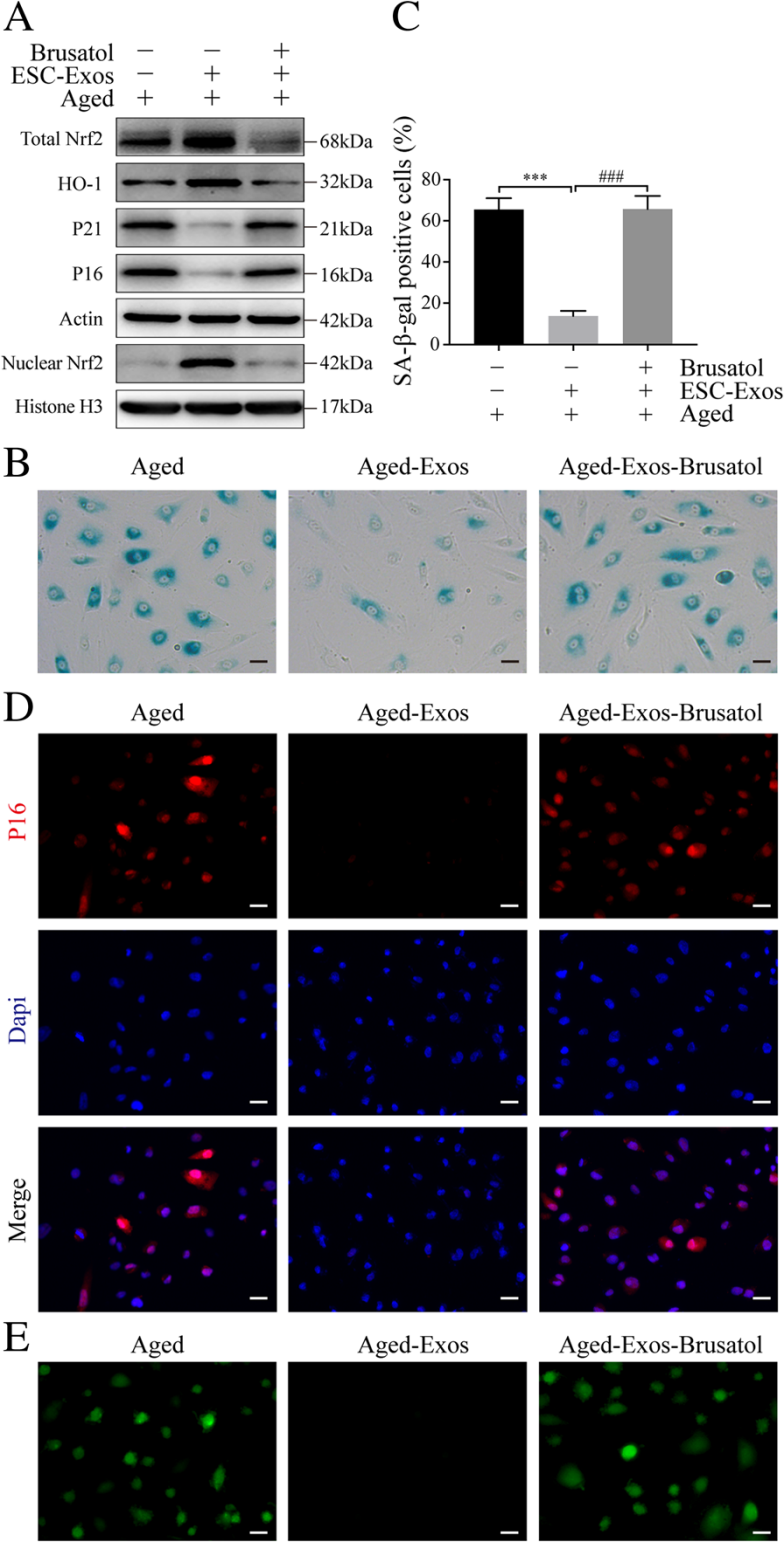
ESC-Exos rejuvenate endothelial senescent cells through activating Nrf2. Aged HUVECs were treated with ESC-Exos or co-treated with ESC-Exos and Nrf2 inhibitor (Brusatol), while aged HUVECs without treatment were set as control. **a** Western blot analysis of Total Nrf2, Nuclear Nrf2, HO1, P21, and P16 protein levels. **b**, **c** SA-β-gal kit was used to evaluate the SA-β-gal activity and percentages of SA-β-gal-positive cells were quantified. *n* = 3 per group. ***P < 0.001 Aged-Exos versus Aged group; ^###^*P* < 0.001 Aged-Exos-Brusatol versus Aged-Exos group. Scale bar, 50 μm. **d** IF staining against P16 was performed to assess the expression level of P16. *n* = 3 per group. Scale bar, 50 μm. **e** ROS levels were determined by green fluorescent intensity after cells were labeled with DCFH-DA. *n* = 3 per group. Scale bar, 50 μm

To examine whether Nrf2 upregulation contributes to the rejuvenative effects of ESC-Exos, aged HUVECs were cotreated with the Nrf2 inhibitor (Brusatol) and ESC-Exos. Western blot analysis showed that Brusatol incubation blocked ESC-Exos-mediated upregulation of Nrf2 and downregulation of P16 and P21 (Fig. 7a). ESC-Exos failed to reduce the SA-훽-gal activity in aged HUVECs when co-treated with Brusatol (Fig. 7b, c), as well as the P16 expression determined by IF staining (Fig. 7d). And, the downregulation of ROS activity mediated by ESC-Exos was obviously abolished by Brusatol treatment (Fig. 7e). Then, we checked the role of Nrf2 in ESC-Exos induced the angiogenic phenotypes of aged HUVECs. As shown in Additional file 4: Figure S4 and Additional file 5: Figure S5, the improved proliferative ability, migratory capacity, and tubule formation of senescent HUVECs by ESC-Exos stimulation were abolished by Brusatol co-treatment. These results suggested that ESC-Exos rejuvenate endothelial senescence and restores aging-related angiogenic dysfunction through Nrf2 activation.

### ESC-Exos activate Nrf2 by downregulating the expression of Keap1 via transferring miR-200a

**Fig. 8:**
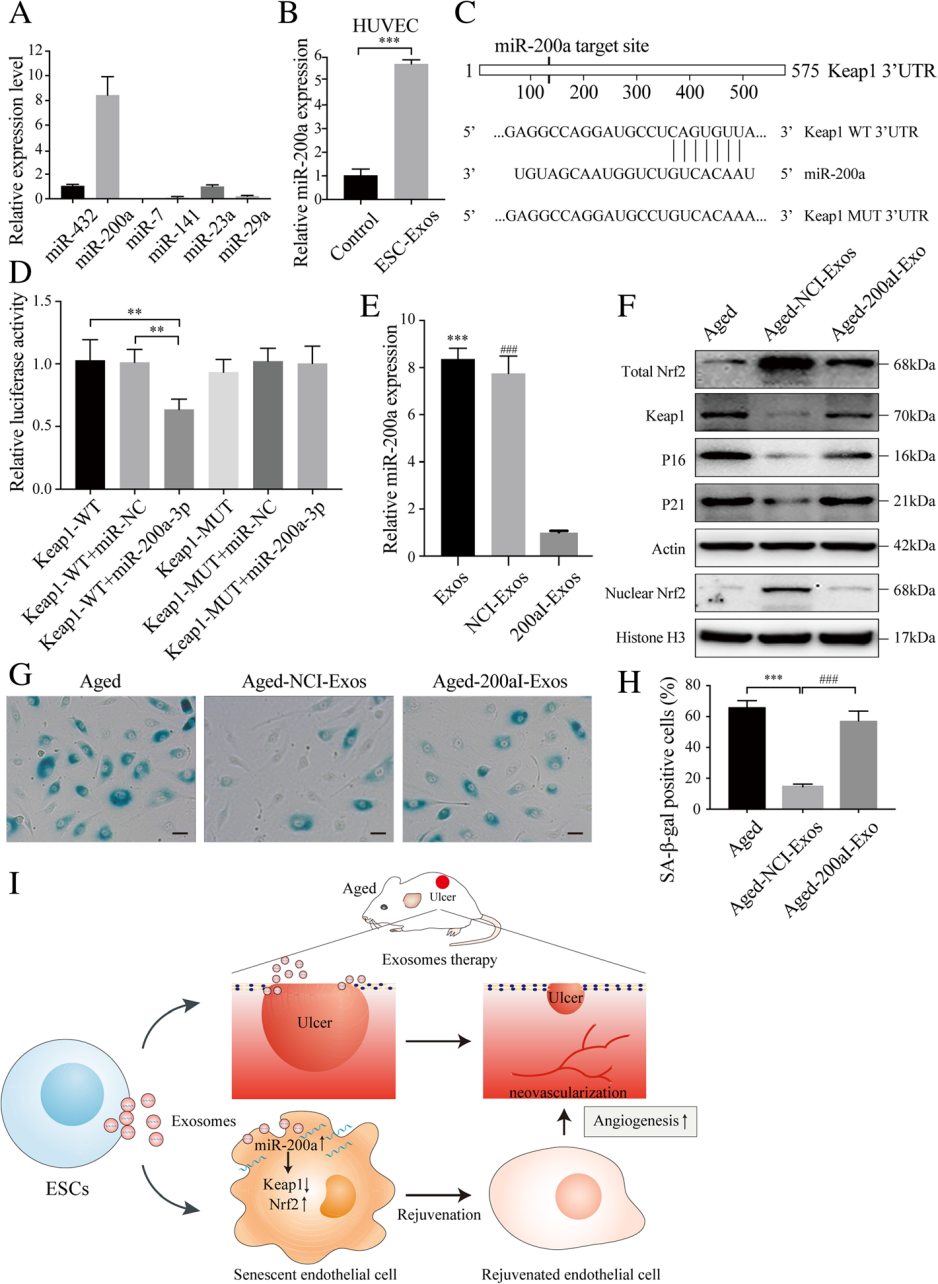
ESC-Exos activated Nrf2 signaling by downregulating the expression of Keap1 via transferring miR-200a. **a** Detection of the expression of the indicated miRNAs in ESC-Exos by qRT-PCR analysis. **b** Aged HUVECs incubated with ESC-Exos for 6 h and the expression level of miR-200a were determined by qPCR analysis. *n* = 3 per group. ***P < 0.001. **c** The Keap1 3′-UTR contains one putative miR-200a binding site. The first eight nucleotides of miR-200a are complementary to the binding site in the 3′-UTR. All of these eight nucleotides were mutated to abrogate miR-200a binding. **d** Dual-luciferase reporter assay of miR-200a with 3′-UTR vectors (WT or MUT) of human Keap1 in HUVECs was performed. We found that miR-200a directly targets the 3′-UTR of Keap1. **e** Levels of miR-200a in exosomes from treated ESCs were analyzed by qRT-PCR. ***P < 0.001 Exos versus NCI-Exo; ^###^*P* < 0.001 NCI-Exo versus 200aI-Exos. **f** Western blot analysis of Nrf2, Keap1, P21, and P16 protein levels. Aged HUVECs were treated with NCI-ESC-Exos or 200aI-ESC-Exos, while aged HUVECs without treatment were set as the control. *n* = 3 per group. **g**, **h** SA-β-gal staining. The SA-β-gal activity and percentages of SA-β-gal-positive cells were quantified. *n* = 3 per group. ***P < 0.001 Aged-NCI-Exos versus Aged group; ^###^*P* < 0.001 Aged-NCI-Exos versus Aged-200aI-Exos. Scale bar, 50 μm. **i** Schematic diagram depicts rejuvenative effects of exosomes derived from embryonic stem cells. Exosomes secreted by ESCs induce enhanced angiogenesis and promoted pressure ulcer repair in aged mice. ESC-Exos-delivered miR-200a rejuvenates senescent endothelial cells by downregulating Keap1 and recovering Nrf2 activation

We further investigated the mechanism by which ESC-Exos activated Nrf2 in senescent endothelial cells. Previous studies reported that Keap1 is a negative regulatory protein of Nrf2 and downregulation of Keap1 has been reported to contribute to Nrf2 activation [33, 34]. The expression of Keap1 is found to be upregulated with age [35]. Our results also show that chronic D-gal treatment led to increased expression of Keap1 in HUVECs, and ESC-Exos treatment reduced Keap1 expression in senescent HUVECs (Fig. 6c), indicating that ESC-Exos may activate Nrf2 by repressing Keap1. Recent studies have reported that miRNAs encapsulated in exosomes can be transferred to recipient cells and modulate their function through regulating gene expression post-transcriptionally [15, 36]. Several miRNAs including miR-7, miR-200a, miR-141a, miR-432, miR-29, and miR-23a have been demonstrated to regulate Nrf2 activity by targeting the expression of Keap1. To verify whether Keap1 is downregulated by these miRNAs transferred via ESC-Exos, we examined the levels of these miRNAs by miRNA-specific quantitative real-time PCR (qRT-PCR). We found that miR-200a was highly enriched in ESC-Exos (Fig. 8a). The levels of miR-200a in aged HUVECs were upregulated after ESC-Exos incubation (Fig. 8b), which is coincident with the downregulation of Keap1 (Fig. 6c). Next, a dual-luciferase reporter assay was performed to identify whether miR-200a targets the 3′-UTR of Keap1 mRNA. HUVECs co-transfected with pMir-Glo vector containing 3′-UTR-WT regions of Keap1 or miR-200a mimics showed significantly less relative luciferase activity than their controls, while mutation of the potential binding sites abolished this effect (Fig. 8c-d). These results indicate that miR-200a can be transferred into HUVECs to regulate Keap1 expression.

To evaluate the role of miR-200a in the ESC-Exos-mediated rejuvenation of senescent HUVECs, the miR-200a level in ESC-Exos was downregulated (Fig. 8e), and aged HUVECs treated with 200aI-ESC-Exos. The results of miR-200a knockdown show that ESC-Exos failed to reduce Keap1 expression and upregulate Nrf2 (Fig. 8f). Moreover, miR-200a downregulation blocked the rejuvenative effects of ESC-Exos on aging phenotypes of senescent endothelial cells (Fig. 8g-h). Then, we checked the role of exosomes transferred miR-200a in ESC-Exos induced the angiogenic phenotypes of aged HUVECs. As shown in Additional file 6: Figure S6, the improved proliferative ability, migratory capacity, and tubule formation of senescent HUVECs by ESC-Exos stimulation were abolished by miR-200a knockdown. These findings suggest that miR-200a is one of the crucial mediators in ESC-Exos-induced rejuvenation of endothelial senescent cells by downregulating Keap1 expression and thereby activating Nrf2.

## Discussion

Aging is an inevitable biological process. Senescent cells accumulating in various tissues during aging contribute to organismal aging and disrupt wound healing after injury [1, 2]. Pressure ulcer wounds, particularly for elderly populations, have been reported to heal poorly, because of aging-related changes in skin tissue [3, 4, 7]. Stem cells, holding great therapeutic promise for various aging-related disorders, have been demonstrated to accelerate wound healing in aged mice, though the underlying mechanisms remain unclear [7]. And, whether stem cell-derived exosomes could promote wound healing in aged individuals is barely reported. In this study, ESC-Exos were locally applied to treat pressure ulcer wounds in an aged mice model induced by D-gal treatment. We found that chronic ESC-Exos treatment effectively rejuvenate endothelial cell senescence and promote angiogenesis, enhancing wound healing.

Angiogenesis, the process by which new blood vessels are formed, plays vital roles in wound healing [3]. We have previously reported that the underlying mechanisms of tissue recovery after exosome treatment partly involve exosome-mediated pro-angiogenesis effects, including cutaneous wound healing, ischemic hindlimb injury repair, and bone regeneration [18–20]. Vascular endothelial cells are major effector cells in the angiogenic process of pressure ulcer healing; aging-related endothelial dysfunction and impaired angiogenesis likely contribute to delayed wound healing in the elderly [4]. And applying anti-aging agents to wound beds could rejuvenate cutaneous cell viability, promote neo-vascularization, and enhance wound healing in aged skin [4]. Thus, rejuvenating endothelial senescent cells and reversing aging-associated angiogenic dysfunction seem to comprise a promising therapeutic approach for wound healing in aged individuals. In our study, we found that the number of senescent endothelial cells at wound beds was significantly reduced after chronic application of ESC-Exos. Also, D-gal-induced senescence in HUVECs was used to evaluate the rejuvenative effects of ESC-Exos in vitro; we found that endothelial senescence is correlated with a decrease in endothelial function (e.g., proliferative, migrative, and tube formation capacities), which is in accordance with the results of previous research [5, 6]. Moreover, chronic ESC-Exos treatment could reduce the aging hallmarks and recover the compromised function. Thus, the therapeutic effects of ESC-Exos on pressure ulcer healing in aged skin may be mainly attributed to their function in rejuvenating endothelial senescent cells and recovering angiogenic function.

Oxidative stress is widely believed to contribute to the aging process [37]. Nrf2 signaling is one of the main cellular defense mechanisms against oxidative stress, but it shows decreased activity in senescence. Also, the basal Nrf2 protein expression levels were found to be downregulated in cells from older donors when compared to cells from young adults [28, 37–39]. Several studies have demonstrated that Nrf2 activity is an essential modulator of species longevity; when Nrf2 expression was suppressed in “young” cells, the cellular function was obviously impaired, while Nrf2 enhancement is sufficient to counteract cellular senescence and render them similar to young cells [30, 32, 40, 41]. Besides, Nrf2 has been demonstrated to play a crucial role in preserving the functional integrity of endothelial cells, and Nrf2 dysfunction is a potential mechanism underlying impaired angiogenesis and microvascular rarefaction in aging [5]. In our study, chronic D-gal treatment resulted in increased oxidative stress. ESC-Exos treatment could restore the oxidative stress balance and reduce ROS intensity. The expression levels of Nrf2 and HO1 (an Nrf2 downstream gene) could be restored after ESC-Exos treatment, which was downregulated after chronic D-gal incubation. Further study revealed that, upon co-treatment with the Nrf2 inhibitor Brusatol, ESC-Exos failed to rejuvenate aged HUVECs and restore impaired angiogenesis. These results suggest that ESC-Exos reverse endothelial senescence through activating Nrf2 expression, but the question how ESC-Exos upregulate Nrf2 expression still requires further investigation.

As for Nrf2 signaling, Keap1 directly leads to continual ubiquitination and subsequent degradation of Nrf2 in the cytoplasm, maintaining it at basal levels [35]. However, significantly increased Keap1 levels were found in aging cells, and the overexpressed Keap1 was considered to impact Nrf2 activity in the elderly [28, 35, 37]. Conversely, increased levels of Nrf2 activity in long-lived species are partially due to reduced expression of Keap1 [40]; downregulation of the expression of Keap1 through several strategies has been demonstrated to activate Nrf2 signaling and exert therapeutic effects [33, 34, 42]. In our study, chronic D-gal incubation resulted in increased expression of Keap1 in HUVECs, and ESC-Exos treatment significantly reduced the levels of Keap1, so it will be very interesting to study how ESC-Exos downregulate Keap1 expression and how this results in Nrf2 upregulation. It has been shown that exosomes act as a delivery system to transfer miRNAs to recipient cells, altering the gene expression and bioactivity of recipient cells by inhibiting mRNA translation or by targeting mRNA for degradation [43]. Accumulating evidence suggests that many miRNAs may regulate Nrf2 activity by targeting the expression of Keap1 [33, 42, 44]. Therefore, we detected the miRNAs in ESC-Exos; we found that miR-200a was the most highly expressed miRNA among the detected miRNAs. Further study demonstrated that upon the miR-200a level in ESC-Exos was downregulated, ESC-Exos almost failed to downregulate the expression of Keap1 and to activate Nrf2 expression. The rejuvenative effect of ESC-Exos was abolished by miR-200a downregulation, although not totally. These findings suggest that miR-200a is one of the crucial mediators in ESC-Exos-induced rejuvenation of endothelial senescent cells by downregulating Keap1 expression and activating Nrf2. However, it should be noted that the effects of ESC-Exos on Nrf2 activation and HUVEC senescence were not entirely abolished by miR-200a downregulation in ESC-Exos, suggesting that other mechanisms may be involved in these processes. This matter requires further investigation. Another limitation of our study is that we do not have in vivo data that show the role of miR-200a in the ESC-Exos-mediated rejuvenation of senescent endothelial cells and the acceleration of wound healing.

## Conclusions

In summary, we for the first time demonstrate that the ESC-Exos accelerate wound healing process and promote local angiogenesis at wound site in aged mice by rejuvenating endothelial senescence. ESC-Exos exerts the anti-aging effects through transferring miR-200a to senescent endothelial cells and activating Nrf2 signaling, which is one of the important pathways involve in anti-aging. Our work indicates that ESC-Exos may function like their parental embryonic stem cells and play important roles in anti-aging therapy and regenerative medicine by transferring the encapsulated bioactive molecules to target cells. ESC-Exos may be a novel cell-free therapeutic tool for aging-related diseases.

## Additional files

Additional file 1: **Figure S1.** D-gal-induced skin aging model. (A) General view of mice after D-gal treatment. (B) Representative images of young and D-gal-induced aging skin stained with H&E. *n* = 3 per group. Scale bar: 50 μm. (C) Thickness of epidermis, dermis, subcutaneous tissue, and muscle layers of young and D-gal-induced aging skin is shown. *n* = 3 per group. ***P < 0.001. (D) IF staining against P16 was performed to assess the expression levels of P16. *n* = 3 per group. Scale bar: 50 μm. (E) Oxidative stress levels were evaluated by measuring the activity of MDA, SOD, GSH-Px, and CAT. *n* = 3 per group. ***P < 0.001. (F) DIO labeled exosomes were locally applied onto the wound beds, and wound sites without labeled exosomes administration were set as control. Wound sites were observed with fluorescence microscopy on 24 h after DIO-labeled exosomes local application. The results revealed that ESC-Exos could permeate through pressure ulcer wound beds. *n* = 3 per group. Scale bar: 200 μm. (TIF 4809 kb)

Additional file 2: **Figure S2.** ESC-Exos could be internalized into aged HUVECs and reverse endothelial senescence. (A) Fluorescence microscopy analysis revealed that DIO-labeled ESC-Exos could be internalized by aged HUVECs. n = 3 per group. Scale bar: 50 μm. (B) SA-β-gal staining. ESC-Exos could reverse HUVEC senescence in a time-dependent manner. *n* = 3 per group. Scale bar: 50 μm. *P < 0.05; **P < 0.01; ***P < 0.001. (TIF 5243 kb)

Additional file 3: **Figure S3.** ESC-Exos can ameliorate aging-related proliferative dysfunction of HUVECs induced by D-gal. HUVECs were treated with 10 g/L D-gal to induce senescence, and aged HUVECs were then treated with 1 × 10^10^ particles/mL ESC-Exos or PBS, while young HUVECs (without D-gal treatment) were set as control. Proliferation of HUVECs was evaluated with the CCK8 kit from day 1 to day 5. ***P < 0.001; **P < 0.01 Aged versus Young group; ^###^*P* < 0.001; ^##^*P* < 0.01 Aged-Exos versus Aged group. (TIF 239 kb)

Additional file 4: **Figure S4.** The Nrf2 inhibitor Brusatol abolished the rejuvenative effect of ESC-Exos on aging-related angiogenic dysfunction of HUVECs. Aged HUVECs were treated with ESC-Exos or co-treated with ESC-Exos and Brusatol, while aged HUVECs without treatment were set as control. (A) IF staining against Ki67 was performed to assess the proliferative capacity of HUVECs. Scale bar: 50 μm. (B) Quantification of the number of ki67-positive cells in (A). *n* = 3 per group. ***P < 0.001 Aged-Exos versus Aged group; ^###^*P* < 0.001 Aged-Exos-Brusatol versus Aged-Exos group. Wound healing assay (C–D) (scale bar: 100 μm) and transwell assay (E–F) (scale bar: 100 μm) were performed to determine the migratory ability of HUVECs. *n* = 3 per group. ***P < 0.001 Aged-Exos versus Aged group; ^###^*P* < 0.001 Aged-Exos-Brusatol versus Aged-Exos group. (G) Representative images of the tube formation assay in three groups. Scale bar: 200 μm. (H) Quantitative analyses of the total tube length and branch points. *n* = 3 per group. ***P < 0.001 Aged-Exos versus Aged group; ^###^*P* < 0.001 Aged-Exos-Brusatol versus Aged-Exos group. (TIF 8099 kb)

Additional file 5: **Figure S5.** The Nrf2 inhibitor Brusatol abolished the rejuvenative effect of ESC-Exos on recovering the compromised proliferative ability of aged HUVECs. Aged HUVECs were treated with ESC-Exos or co-treated with ESC-Exos and Brusatol, while aged HUVECs without treatment were set as the control. Proliferation of HUVECs was evaluated with the CCK8 kit from day 1 to day 5. *P < 0.05; **P < 0.01; ***P < 0.001 Aged-Exos versus Aged group; ^##^*P* < 0.01; ^###^*P* < 0.001 Aged-Exos-Brusatol versus Aged-Exos group. (TIF 250 kb)

Additional file 6: **Figure S6.** The miR-200a downregulation abolished the rejuvenative effect of ESC-Exos on aging-related angiogenic dysfunction of HUVECs. Aged HUVECs were treated with NCI-ESC-Exos or 200aI-ESC-Exos, while aged HUVECs without treatment were set as control. (A) IF staining against Ki67 was performed to assess the proliferative capacity of HUVECs. Scale bar: 50 μm. (B) Quantification of the number of ki67-positive cells in (A). *n* = 3 per group. ***P < 0.001 Aged-NCI-Exos versus Aged group; ^###^*P* < 0.001 Aged-NCI-Exos versus Aged-200aI-Exos. Wound healing assay (C–D) (scale bar: 100 μm) and transwell assay (E–F) (scale bar: 100 μm) were performed to determine the migratory ability of HUVECs. *n* = 3 per group. ***P < 0.001 Aged-NCI-Exos versus Aged group; ^###^*P* < 0.001 Aged-NCI-Exos versus Aged-200aI-Exos. (G) Representative images of the tube formation assay in three groups. Scale bar: 200 μm. (H) Quantitative analyses of the total tube length and branch points. *n* = 3 per group. ***P < 0.001 Aged-NCI-Exos versus Aged group; ^###^*P* < 0.001 Aged-NCI-Exos versus Aged-200aI-Exos. (TIF 8136 kb)

Additional file 7: **Table S1.** Sequences used for qRT-PCR (DOCX 13 kb)

## Abbreviations

ECM: Endothelial cell medium
ESCs: Embryonic stem cells
FBS: Fetal bovine serum
H&E: Hematoxylin eosin
HUVECs: Human umbilical vein endothelial cells
Keap1: Kelch-like ECH-associated protein 1
Nrf2: Nuclear factor (erythroid-derived 2)-like 2
ROS: Reactive oxygen species
SA-β-gal: Senescence-associated β-galactosidase
TEM: Transmission electron microscopy
