# Supplementary figures and images for: Human embryonic stem cell-derived exosomes promote pressure ulcer healing in aged mice by rejuvenating senescent endothelial cells
Source: Stem Cell Res Ther. 2019 May 21;10:142. doi: 10.1186/s13287-019-1253-6 (PMC6528288; doi:10.1186/s13287-019-1253-6)

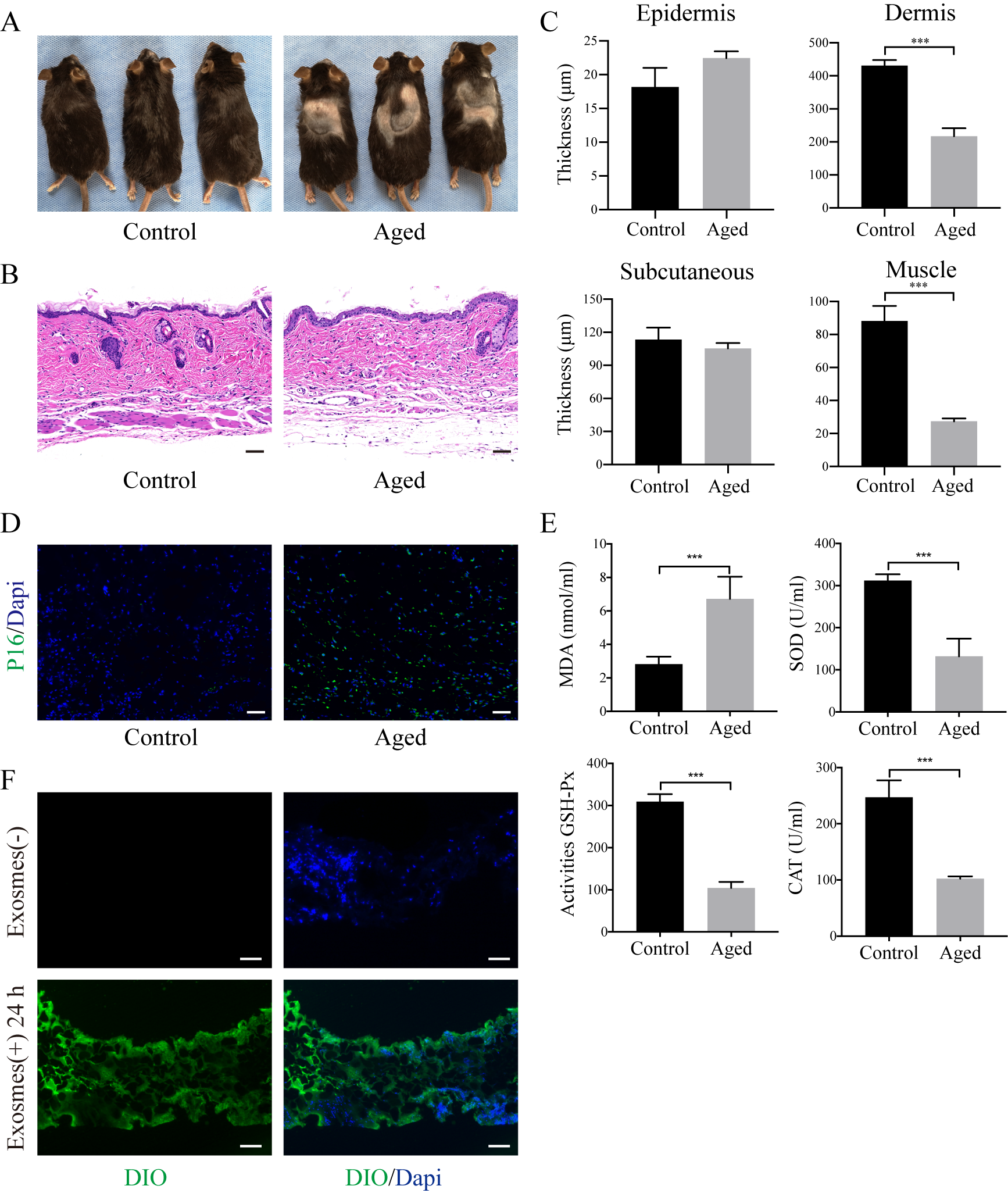

Supplement: Supplementary file 1 — Figure S1. D-gal-induced skin aging model. (A) General view of mice after D-gal treatment. (B) Representative images of young and D-gal-induced aging skin stained with H&E. n = 3 per group. Scale bar: 50 μm. (C) Thickness of epidermis, dermis, subcutaneous tissue, and muscle layers of young and D-gal-induced aging skin is shown. n = 3 per group. ***P < 0.001. (D) IF staining against P16 was performed to assess the expression levels of P16. n = 3 per group. Scale bar: 50 μm. (E) Oxidative stress levels were evaluated by measuring the activity of MDA, SOD, GSH-Px, and CAT. n = 3 per group. ***P < 0.001. (F) DIO labeled exosomes were locally applied onto the wound beds, and wound sites without labeled exosomes administration were set as control. Wound sites were observed with fluorescence microscopy on 24 h after DIO-labeled exosomes local application. The results revealed that ESC-Exos could permeate through pressure ulcer wound beds. n = 3 per group. Scale bar: 200 μm. (TIF 4809 kb) [file 13287_2019_1253_MOESM1_ESM.tif]

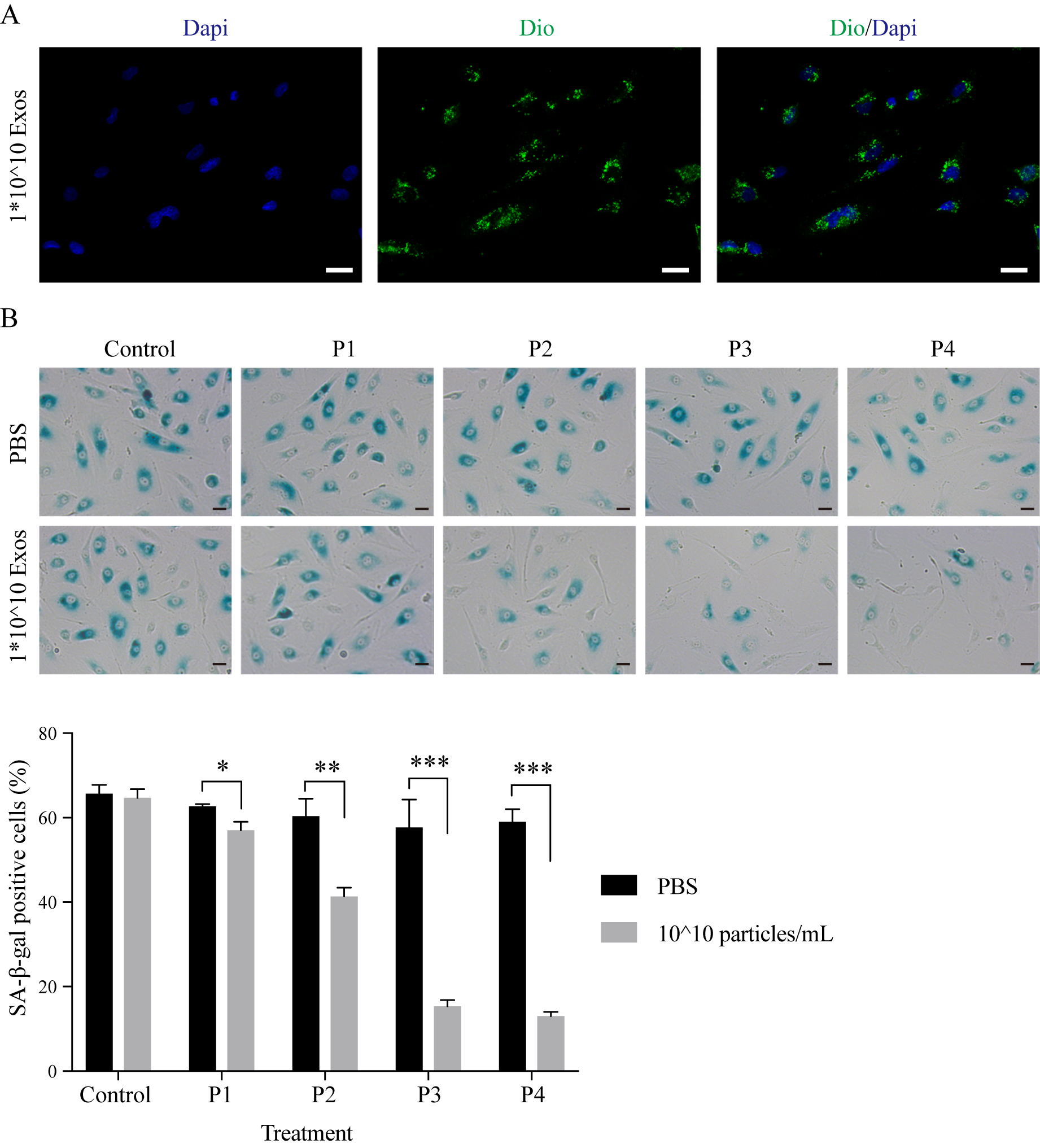

Supplement: Supplementary file 2 — Figure S2. ESC-Exos could be internalized into aged HUVECs and reverse endothelial senescence. (A) Fluorescence microscopy analysis revealed that DIO-labeled ESC-Exos could be internalized by aged HUVECs. n = 3 per group. Scale bar: 50 μm. (B) SA-β-gal staining. ESC-Exos could reverse HUVEC senescence in a time-dependent manner. n = 3 per group. Scale bar: 50 μm. *P < 0.05; **P < 0.01; ***P < 0.001. (TIF 5243 kb) [file 13287_2019_1253_MOESM2_ESM.tif]

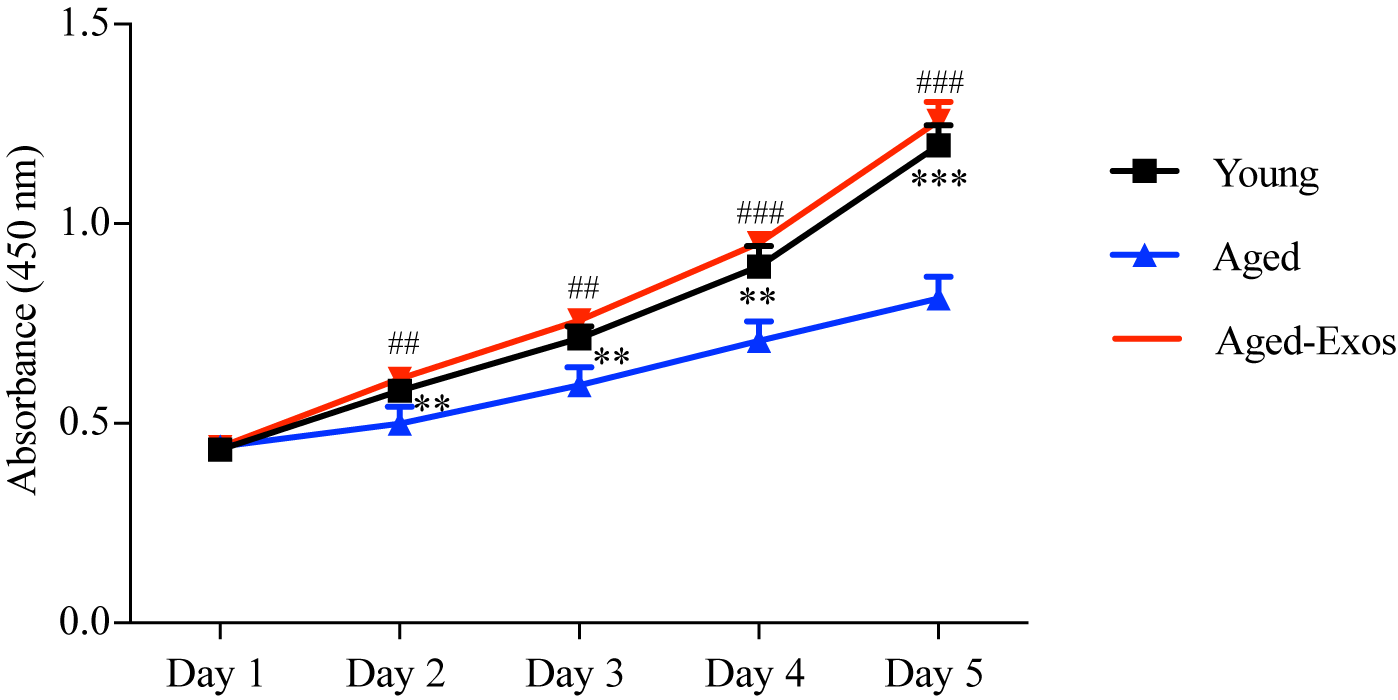

Supplement: Supplementary file 3 — Figure S3. ESC-Exos can ameliorate aging-related proliferative dysfunction of HUVECs induced by D-gal. HUVECs were treated with 10 g/L D-gal to induce senescence, and aged HUVECs were then treated with 1 × 10^10^ particles/mL ESC-Exos or PBS, while young HUVECs (without D-gal treatment) were set as control. Proliferation of HUVECs was evaluated with the CCK8 kit from day 1 to day 5. ***P < 0.001; **P < 0.01 Aged versus Young group; ###P < 0.001; ##P < 0.01 Aged-Exos versus Aged group. (TIF 239 kb) [file 13287_2019_1253_MOESM3_ESM.tif]

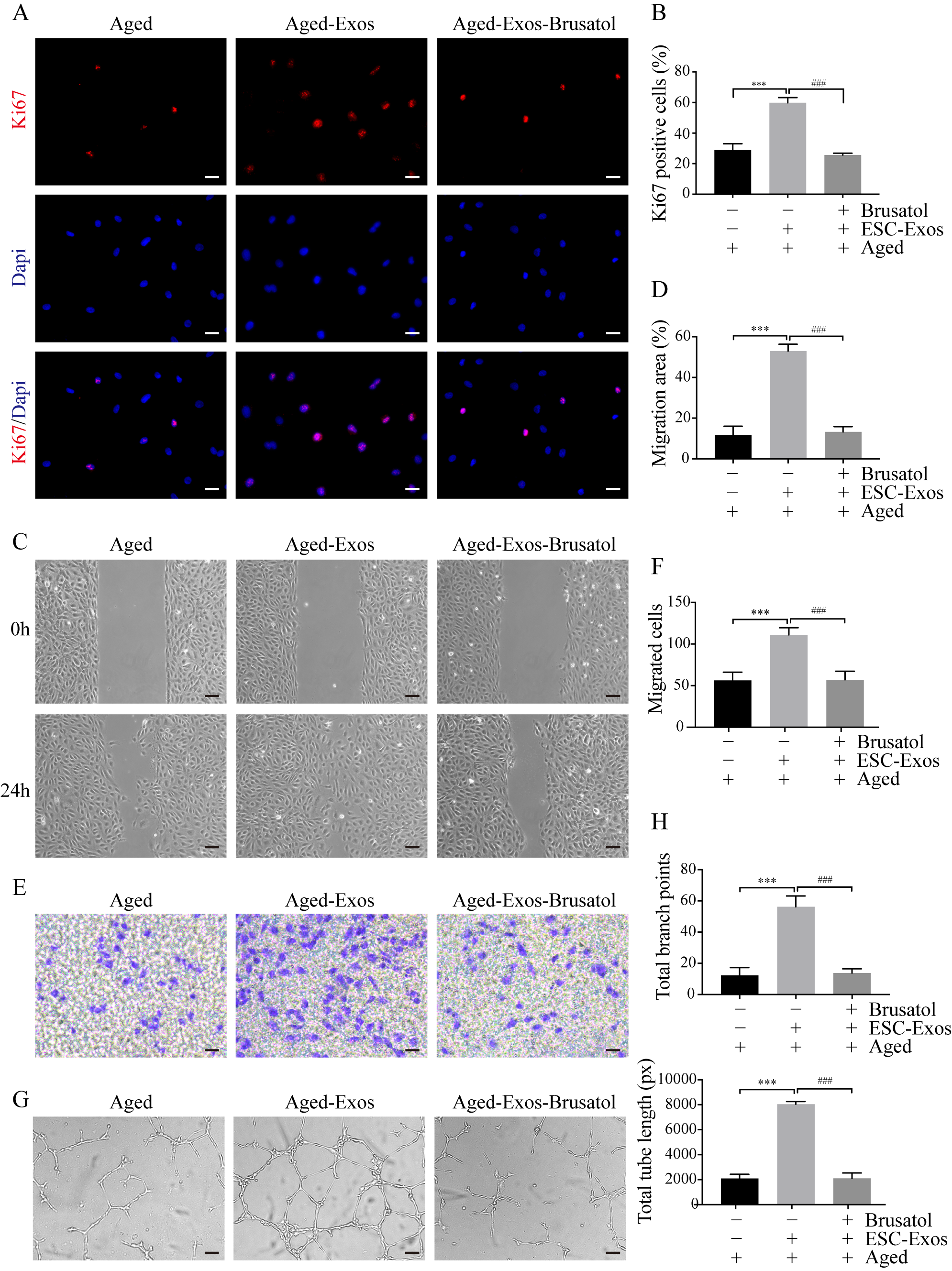

Supplement: Supplementary file 4 — Figure S4. The Nrf2 inhibitor Brusatol abolished the rejuvenative effect of ESC-Exos on aging-related angiogenic dysfunction of HUVECs. Aged HUVECs were treated with ESC-Exos or co-treated with ESC-Exos and Brusatol, while aged HUVECs without treatment were set as control. (A) IF staining against Ki67 was performed to assess the proliferative capacity of HUVECs. Scale bar: 50 μm. (B) Quantification of the number of ki67-positive cells in (A). n = 3 per group. ***P < 0.001 Aged-Exos versus Aged group; ###P < 0.001 Aged-Exos-Brusatol versus Aged-Exos group. Wound healing assay (C–D) (scale bar: 100 μm) and transwell assay (E–F) (scale bar: 100 μm) were performed to determine the migratory ability of HUVECs. n = 3 per group. ***P < 0.001 Aged-Exos versus Aged group; ###P < 0.001 Aged-Exos-Brusatol versus Aged-Exos group. (G) Representative images of the tube formation assay in three groups. Scale bar: 200 μm. (H) Quantitative analyses of the total tube length and branch points. n = 3 per group. ***P < 0.001 Aged-Exos versus Aged group; ###P < 0.001 Aged-Exos-Brusatol versus Aged-Exos group. (TIF 8099 kb) [file 13287_2019_1253_MOESM4_ESM.tif]

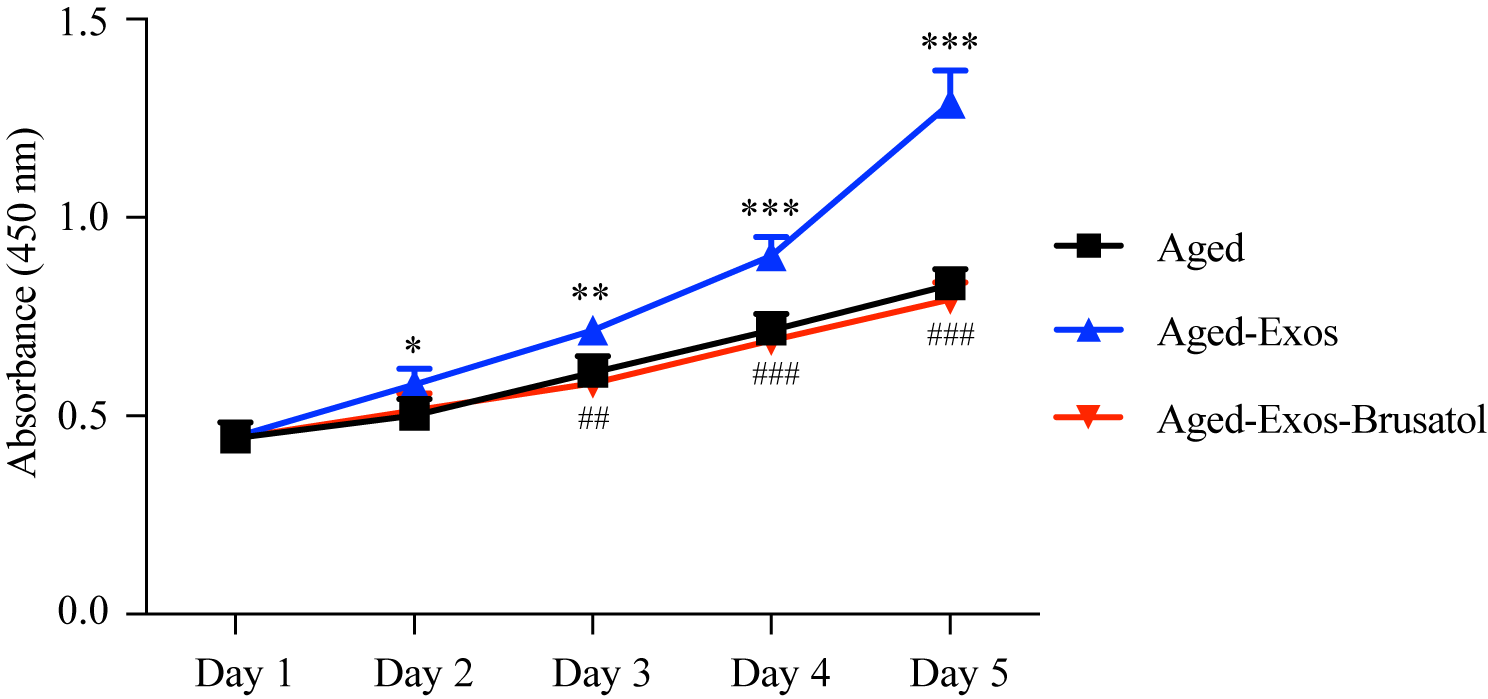

Supplement: Supplementary file 5 — Figure S5. The Nrf2 inhibitor Brusatol abolished the rejuvenative effect of ESC-Exos on recovering the compromised proliferative ability of aged HUVECs. Aged HUVECs were treated with ESC-Exos or co-treated with ESC-Exos and Brusatol, while aged HUVECs without treatment were set as the control. Proliferation of HUVECs was evaluated with the CCK8 kit from day 1 to day 5. *P < 0.05; **P < 0.01; ***P < 0.001 Aged-Exos versus Aged group; ##P < 0.01; ###P < 0.001 Aged-Exos-Brusatol versus Aged-Exos group. (TIF 250 kb) [file 13287_2019_1253_MOESM5_ESM.tif]

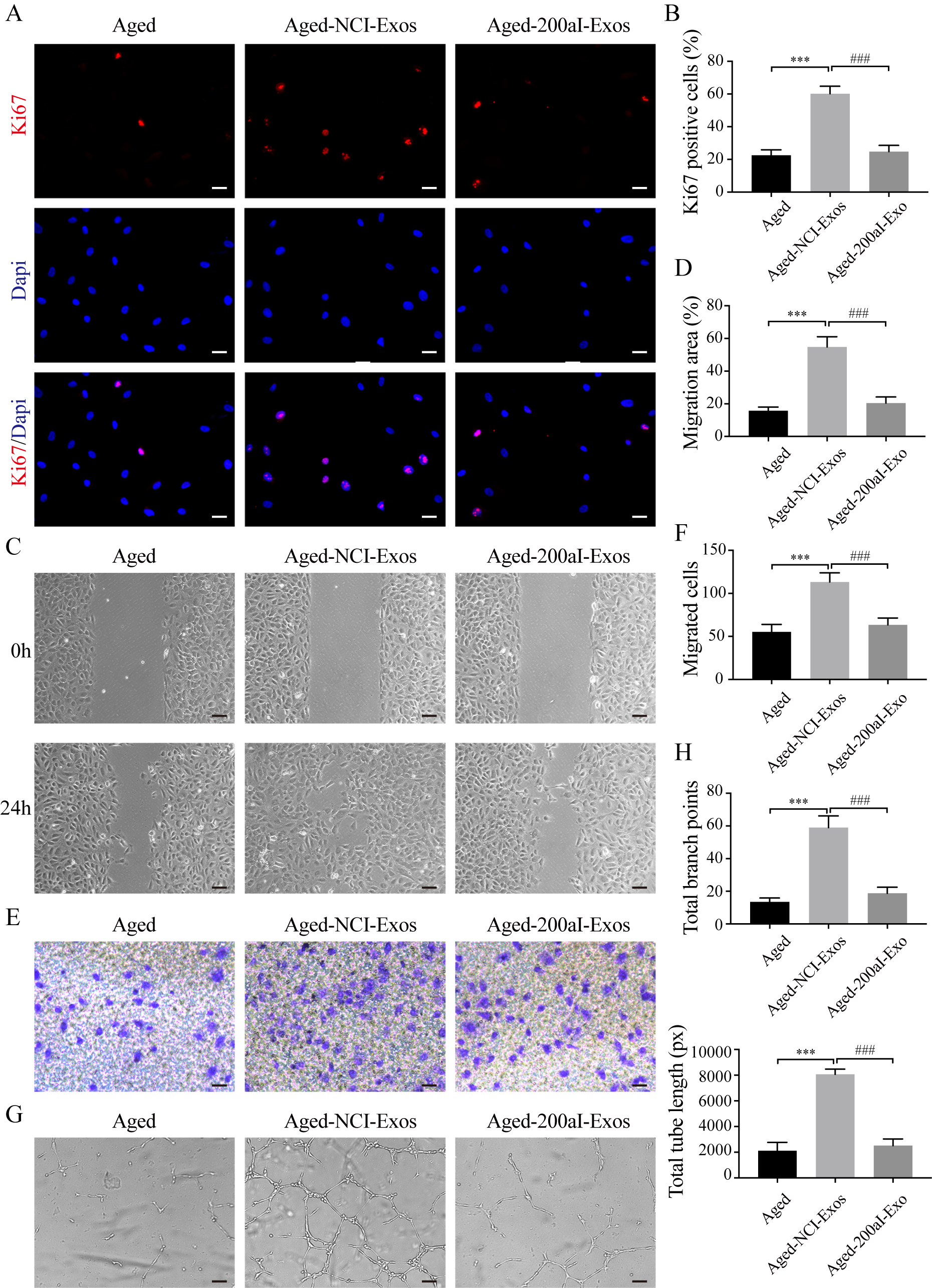

Supplement: Supplementary file 6 — Figure S6. The miR-200a downregulation abolished the rejuvenative effect of ESC-Exos on aging-related angiogenic dysfunction of HUVECs. Aged HUVECs were treated with NCI-ESC-Exos or 200aI-ESC-Exos, while aged HUVECs without treatment were set as control. (A) IF staining against Ki67 was performed to assess the proliferative capacity of HUVECs. Scale bar: 50 μm. (B) Quantification of the number of ki67-positive cells in (A). n = 3 per group. ***P < 0.001 Aged-NCI-Exos versus Aged group; ###P < 0.001 Aged-NCI-Exos versus Aged-200aI-Exos. Wound healing assay (C–D) (scale bar: 100 μm) and transwell assay (E–F) (scale bar: 100 μm) were performed to determine the migratory ability of HUVECs. n = 3 per group. ***P < 0.001 Aged-NCI-Exos versus Aged group; ###P < 0.001 Aged-NCI-Exos versus Aged-200aI-Exos. (G) Representative images of the tube formation assay in three groups. Scale bar: 200 μm. (H) Quantitative analyses of the total tube length and branch points. n = 3 per group. ***P < 0.001 Aged-NCI-Exos versus Aged group; ###P < 0.001 Aged-NCI-Exos versus Aged-200aI-Exos. (TIF 8136 kb) [file 13287_2019_1253_MOESM6_ESM.tif]
