# Supplementary material for: Human embryonic stem cell-derived exosomes promote pressure ulcer healing in aged mice by rejuvenating senescent endothelial cells
Source: Stem Cell Res Ther. 2019 May 21;10:142. doi: 10.1186/s13287-019-1253-6 (PMC6528288; doi:10.1186/s13287-019-1253-6)
Supplement: Supplementary file 7 — Table S1. Sequences used for qRT-PCR (DOCX 13 kb) [file 13287_2019_1253_MOESM7_ESM.docx]

| **Additional file 7: Table S1. Sequences used for qRT-PCR** | | |
| --- | --- | --- |
| Gene name | Reverse transcription primer | |
| miR-200a | GTTGGCTCTGGTGCAGGGTCCGAGGTATTCGCACCAGAGCCAACACATCG | |
| miR-7 | GTTGGCTCTGGTGCAGGGTCCGAGGTATTCGCACCAGAGCCAACAAC | |
| miR-141 | GTTGGCTCTGGTGCAGGGTCCGAGGTATTCGCACCAGAGCCAACCATC | |
| miR-432 | GTTGGCTCTGGTGCAGGGTCCGAGGTATTCGCACCAGAGCCAACAGACA | |
| miR-23a | GTTGGCTCTGGTGCAGGGTCCGAGGTATTCGCACCAGAGCCAACGGAAA | |
| miR-29a | GTTGGCTCTGGTGCAGGGTCCGAGGTATTCGCACCAGAGCCAACTAACC | |
| U6 | GTTGGCTCTGGTGCAGGGTCCGAGGTATTCGCACCAGAGCCAACGAATTTGC | |
|  | Forward primer (5’ ➝ 3’) | Reverse primer (5’ ➝ 3’) |
| miR-200a | TAACACTGTCTGGTAAC | GTGCAGGGTCCGAGGT |
| miR-7 | TGGAAGACTAGTGATTTT | GTGCAGGGTCCGAGGT |
| miR-141 | TAACACTGTCTGGTAAAG | GTGCAGGGTCCGAGGT |
| miR-432 | CTGGATGGCTCCTCCA | GTGCAGGGTCCGAGGT |
| miR-23a | ATCACATTGCCAGGGA | GTGCAGGGTCCGAGGT |
| miR-29a | TAGCACCATCTGAAATC | GTGCAGGGTCCGAGGT |
| U6 | CCTGCTTCGGCAGCACA | GTGCAGGGTCCGAGGT |
